# Structure Revision of Penipacids A–E Reveals a Putative New Cryptic Natural Product, *N*-aminoanthranilic Acid, with Potential as a Transcriptional Regulator of Silent Secondary Metabolism

**DOI:** 10.3390/md20060339

**Published:** 2022-05-24

**Authors:** Zeinab G. Khalil, Sarani Kankanamge, Robert J. Capon

**Affiliations:** Institute for Molecular Bioscience, The University of Queensland, St Lucia, QLD 4072, Australia; z.khalil@uq.edu.au (Z.G.K.); s.kankanamge@imb.uq.edu.au (S.K.)

**Keywords:** *N*-aminoanthranilic acid, Schiff base adduct, artifact, structure revision, total synthesis, transcriptional regulator, microbioreactor cultivation profiling

## Abstract

Reconsideration of the spectroscopic data for penipacids A–E, first reported in 2013 as the acyclic amidines **1**–**5** from the South China deep sea sediment-derived fungus *Penicillium paneum* SD-44, prompted a total synthesis structure revision as the hydrazones **6**–**10**. This revision strongly supported the proposition that penipacids A–B (**6**–**7**) were artifact Schiff base adducts of the cryptic (undetected) natural product *N*-aminoanthranilic acid (**11**) with diacetone alcohol, induced by excessive exposure to acetone and methanol under acidic handling conditions. Likewise, the revised structures for penipacids C–D (**8**–**9**) and E (**10**) raise the possibility that they may also be artifact Schiff base adducts of **11** and the media constituents pyruvic acid and furfural, respectively. A review of the natural products literature revealed other Schiff base (hydrazone) natural products that might also be viewed as Schiff base adduct artifacts of **11**. Having raised the prospect that **11** is an undetected and reactive cryptic natural product, we went on to establish that **11** is not cytotoxic to a range of bacterial, fungal or mammalian (human) cell types. Instead, when added as a supplement to microbial cultivations, **11** can act as a chemical cue/transcriptional regulator, activating and/or enhancing the yield of biosynthetic gene clusters encoding for other natural product chemical defenses. This study demonstrates the value of challenging the structure and artifact status of natural products, as a window into the hidden world of cryptic and highly reactive natural products.

## 1. Introduction

**Figure 1:**
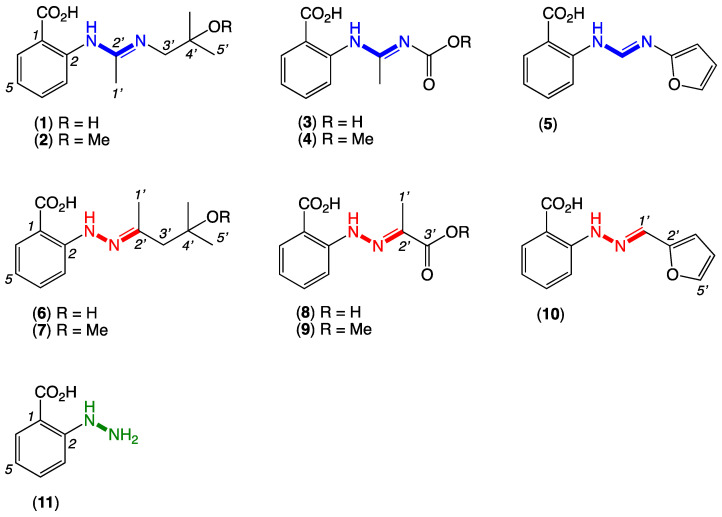
Penipacids A–E with reported (incorrect) **amidine** structures **1**–**5** and revised (correct) **hydrazone** structures **6**–**10**, and the putative cryptic **hydrazine** natural product, *N*-aminoanthranilic acid (**11**).

On reading a 2013 report of the acyclic amidine penipacids A–E (**1**–**5**) from a South China Sea deep sea sediment-derived fungus, *Penicillium paneum* SD-44 [1], we reasoned that the alternate hydrazone structures **6**–**10** were more spectroscopically and biosynthetically plausible (Figure 1). For example, the ^13^C NMR shifts for the amidine sp^2^ C-2’ carbons in **1**–**5** did not appear to be sufficiently deshielded and were more consistent with a hydrazone moiety. Likewise, the biosynthetic precursors needed to assemble the acyclic amidines **1**–**5** lacked precedence and seemed implausible, while those required for the alternate Schiff base hydrazones **6**–**10** were well-precedented and highly plausible. Having raised the prospect that the penipacids were hydrazones, we further speculated some or even all were artifacts generated through Schiff base condensation of a hypothetical and cryptic hydrazine natural product precursor, *N*-aminoanthranilic acid (**11**), with a selection of solvent and media-derived ketones and aldehydes. All these hypotheses were validated by a series of syntheses and spectroscopic comparisons, as outlined below.

## 2. Results

**Figure 2:**
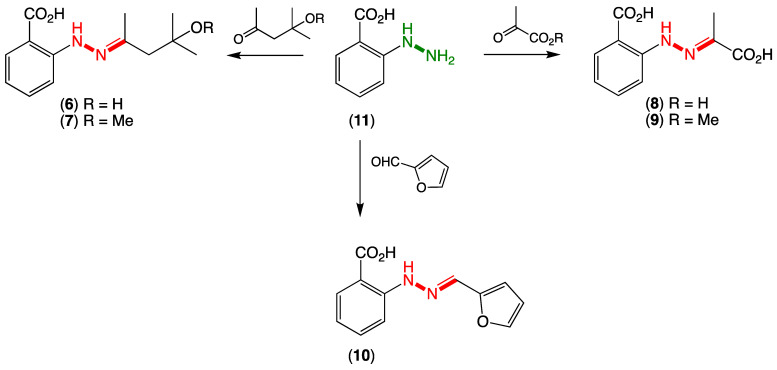
Synthesis of revised hydrazone structures for penipacids A–G (**6**–**10**).

Following an established protocol [2], we first prepared an authentic sample of *N*-aminoanthranilic acid (**11**) and demonstrated that following brief exposure to diacetone alcohol or diacetone alcohol methyl ether under neutral conditions at room temperature, **11** underwent facile conversion to the Schiff base hydrazones **6** and **7**, respectively (Figure 2). Significantly, the hydrazones **6** and **7** were spectroscopically identical to the reported penipacids A and B (Tables S1 and S2 and Figures S9–S15), necessitating structure revision (as noted above). Similarly, following brief exposure to pyruvic acid or methyl pyruvate, **11** underwent facile conversion to the hydrazones **8** and **9**, identical in all respects to penipacids C and D (Tables S3 and S4 and Figures S17–S23). Finally, brief exposure of **11** to furfural resulted in rapid conversion to the hydrazone **10**, spectroscopically identical to penipacid E (Figures S24–S25). Of note, unlike **6**–**9**, efforts to purify **10** resulted only in low recovered yields, most likely due to the more labile nature of the conjugated aromatic hydrazone, which might be expected to be prone to hydrolysis during handling to its constitutent components. Based on the above, we propose that the originally reported structures for penipacids A–E (**1**–**5**) be revised as indicated to **6**–**10**. This revision strongly supported the proposition that penipacids A–B (**6**–**7**) were artifact Schiff base adducts of the cryptic (undetected) natural product *N*-aminoanthranilic acid (**11**) with diacetone alcohol, induced by excessive exposure to acetone and methanol under acidic handling conditions. Likewise, the revised structures for penipacids C–D (**8**–**9**) and E (**10**) at the very least raise the possibility that they, too, may be artifact Schiff base adducts of **11** and the common media constituents pyruvic acid and furfural, respectively. A review of the natural products literature revealed other Schiff base (hydrazone) natural products that might also be viewed as adduct artifacts. Our willingness to speculate on **11** as a cryptic natural product is also informed by our prior experience with the prolinimines A–D (**12**–**15**), which are artifacts of the cryptic fungal natural product *N*-amino-L-prolinimine methyl ester (**16**) [3,4].

**Figure 3:**
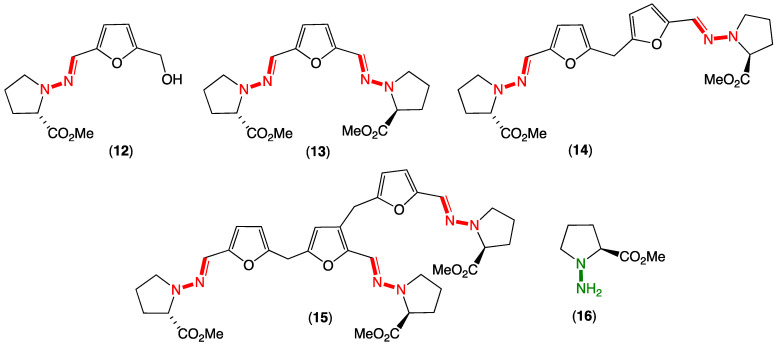
*Evlachovaea* sp. CMB-F563 artifact prolinimines A–D (**12**–**15**) and cryptic natural product *N*-amino-l-proline methyl ester (**16**).

To elaborate briefly on some of our relevant prior experience, in 2018, we reported on unprecedented hydrazone Schiff base prolinimines A–D (**12**–**15**) from a fish gastrointestinal tract-derived fungus, *Evlachovaea* (*Trichoderma*) sp. CMB-F563 (Figure 3) [3]. Alert to the possibility for artifacts, we reported that **14**–**15** were dimer and trimer artifacts of **12**, induced by exposure to acid during isolation and handling and used this to implement a successful biomimetic synthesis. To further expand our understanding of the prolinimines, we embarked on follow-up investigations, and in 2021, we reported that extraction of CMB-F563 cultivations with EtOAc released a new cryptic hydrazino natural product from the fungal mycelia, *N*-amino-l-proline methyl ester (**16**) (Figure 3) [4]. Most importantly, we reported that following solvent extraction, **16** came in contact and underwent rapid Schiff base condensation with the media components 5-hydroxymethylfurfural and 2,5-furandicarboxaldehyde to yield **12**–**13** (Note: 5-hydroxymethylfurfural and 2,5-furandicarboxaldehyde are known thermolysis products generated during autoclaving of carbohydrate-rich media). Thus, and despite their structural novelty, the prolinimines A–D (**12**–**15**) were confirmed to be handling artifacts initially triggered at the point of solvent extraction, with the sole natural product being *N*-amino-l-proline methyl ester (**16**). A key lesson from this study was that despite being artifacts, the prolinimines were useful molecular markers that disclosed the existence of the new, highly cryptic hydrazino natural product **16**. Building on this experience, we have since gone on to apply an in situ Schiff base derivatization strategy to detect **16** and other cryptic water-soluble, small-molecular-weight natural products in fresh culture extracts, including new aminosugars [4]. Overall, the prolinimine experience heightened our appreciation for the significance of artifacts, particularly as potential markers for otherwise chemically reactive and cryptic natural products [4].

The literature account of the production, extraction and purification of penipacids A–E describes exhaustive acetone extraction of a 270 L cultivation of *Pencillium paneum* SD-44, with the concentrated crude extract (70 g) undergoing a series of silica gel (CHCl_3_/MeOH), reversed-phase (MeOH/H_2_O, including with acetic acid) and gel (Sephadex LH-20, MeOH) chromatography. The use of large volumes of acetone brings the very high risk of exposing any natural product(s) to the common aldol condensation artifact diacetone alcohol, while pyruvic acid will also likely be present as a result of media glycolysis, as will furfural as a carbohydrate thermolysis/dehydration product produced during media autoclaving. As noted in a recent review of natural product artifacts [5], exposure to acidic MeOH during fractionation (i.e., silica gel and CHCl_3_/MeOH) can lead to the formation of methyl ethers such as diacetone alcohol methyl ether and esters such as methyl pyruvate. Thus, all five of the carbonyl species used in our total synthesis of penipacids A–E (**6**–**10**) from *N*-aminoanthranilic acid (**11**) (Figure 2) (diacetone alcohol, diacetone alcohol methyl ethert, pyruvic acid, methyl pyruvate and furfural) can be construed as nonfungal in origin and yet present during fractionation and handling of the *Pencillium paneum* SD-44 extract. This raises the possibility that some or all of the penipacids are artifacts of the cryptic but undetected natural product, *N*-aminoanthranilic acid (**11**).

**Figure 4:**
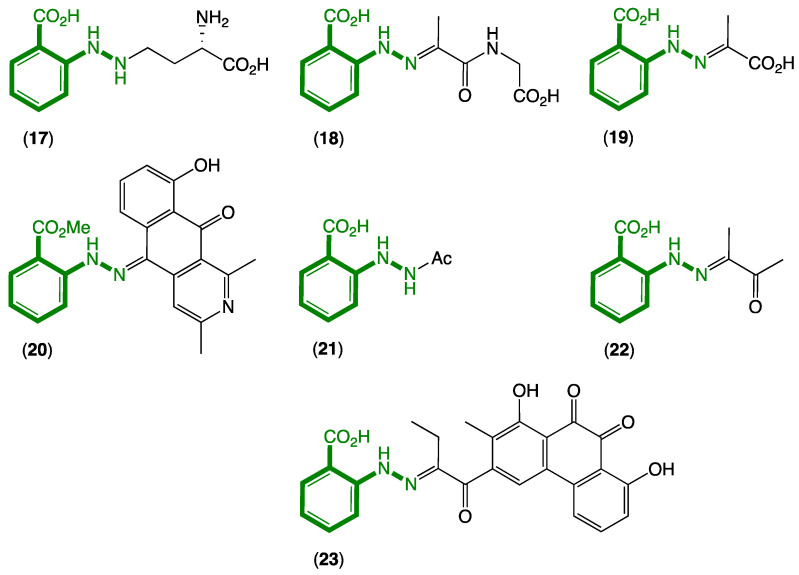
Known natural products **17**–**23** incorporating an *N*-aminoanthranilic acid motif (green).

Although *N*-aminoanthranilic acid (**11**) has yet to be reported as a natural product, there is circumstantial evidence in both the bacterial and fungal natural products literature to support the proposition that it is indeed capable of being a natural product. For example (Figure 4):

(i) In 1978, Minato et al. described a γ-glutamylphenylhydrazine anthglutin (**17**) from a Japanese fungus *Pencillium oxalicum* SANK 10477—a potential conjugate of **11** and l-homoserine [6];
(ii) In 2011, Che et al. reported the phenylhydrazone farylhydrazones A (**18**) and B (**19**) from a Tibetan *Cordyceps*-colonising fungus *Isaria farinose—*potential Schiff base adducts of **11** and pyruvylglycine and pyruvic acid, respectively [7]. Note that the revised structure for penipacid C (**8**) is now identical to farylhydrazone B (**19**);
(iii) In 2012, Ishibashi et al. reported the 2-azoquinone-phenylhydrazine katorazone (**20**) from a Japanese soil-derived *Streptomyces* sp. IFM 11299—a potential Schiff base adduct of **11** with the known fungal metabolite utahmycin A, which was coincidentally reported to be a cometabolite with **20** [8];
(iv) In 2013, Zou et al. reported the first natural occurrence of *N*-acetyl-hydrazinobenzoic acid (**21**) from a Chinese endophytic fungus *Pencillium citrinum—*the *N*-acetate of **11** [9];
(v) In 2016, Zhu et al. reported farylhydrazone C (**22**), along with **19** and **21** from an Antarctic soil-derived *Penicillium* sp. HDN14-431—potentially a Schiff base adduct of **11** with dimethylglyoxal, where the latter is known to be produced during thermal processing of carbohydrate-rich foods and as such could be a media constituent induced during authoclaving [10];
(vi) In 2019, Jiao et al. reported the aromatic polyketide murayaquinone C (**23**) from the ant gut-derived *Streptomyces* sp. NA4286—a potential Schiff base adduct between **11** and a suitably substituted anthraquinone [11].

While knowledge of natural products **17**–**23** incorporating *N*-aminoanthranilic acid moieties goes back to 1978, it is worth noting that there have been no reports of **11** as a natural product, even though **18**–**20** and **22**–**23** could potentially be artifact Schiff base adducts of **11** and well-known carbonyl precursors.

As a water-soluble, small-molecular-weight compound that at a cursory glance might be dismissed as anthranilic acid, it is perhaps not surprising that **11** has gone undetected as a natural product—especially as natural products research is largely focused on organic not water-soluble materials. Notwithstanding, why should we care if **11** is or is not recognized as a natural product? Putting aside that good science requires that we understand and document the real world and not be misled by an induced artificial construct, there are other very good reasons why attention to detail is important. At one level, the prospect that **11** may be a highly chemically reactive cryptic natural product calls into question the status of previously reported “natural product” Schiff bases such as **18**–**20** and **22**–**23**, raising the possibility that some if not all are artifacts induced during extraction and isolation. At another level, given the extent to which science endeavors to understand the biological properties and ecological significance of natural products, the failure to factor in artifacts clouds this perspective and can lead to erroneous conclusions. For example, chemically reactive cryptic natural products could provide a plausible explanation for some of those occasions where significant biological activities detected in fresh extracts fail to survive and/or materialize in isolated natural products—particularly if the isolated artifacts prove to be biologically inactive. Picking up on this latter point and alert to the potential for **11** to conjugate with critically important biological aldehydes/ketones, we initially expected **11** to be a defense metabolite and exhibit a high level of nonspecific cytotoxicity. Hence, we were surprised to observe that **11** did not exhibit any cytotoxic properties (up to 30 μM) against human colorectal (SW620) and lung (NCIH-460) carcinoma cells, nor did it inhibit the growth of the fungal pathogen *Candida albicans* ATCC 10231, the Gram-negative bacterial pathogen *Escherichia coli* ATCC11775 or the Gram-positive bacterial pathogens *Staphylococcus aureus* ATCC25923 and *Bacillus subtilis* ATCC6633 (Figures S26 and S27). While it would appear that the chemical reactivity of **11** is insufficient to cause lethal disruption of cellular processes, we nevertheless speculated some level of disruption may offer a nonlethal survival advantage to the producing organism—perhaps as a chemical cue capable of triggering the transcriptional activation of silent chemical defenses. This view was further informed by prior studies in which we had demonstrated that biologically inactive natural products, such as cyclo-(L-Phe-*trans*-4-hydroxy-L-Pro), can act as transcriptional regulators of silent microbial chemical defenses [12].

**Figure 5:**
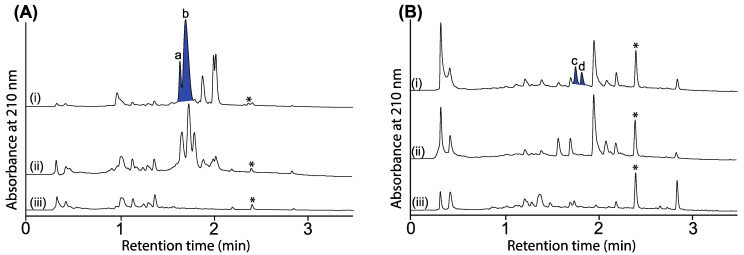
UPLC-DAD (210 nm) chromatograms of cultivations of (**A**) CMB-W003 and (**B**) *Penicillium* sp. CMB-MD22 (i) with and (ii) without *N*-aminoanthranilic acid (30 μM) and (iii) media only, with all transcriptionally activated metabolites highlighted in blue and identified as (a) roquefortine C, (b) oxaline and (c,d) new neobulgarones, and where * is an internal calibrant.

**Figure 6:**
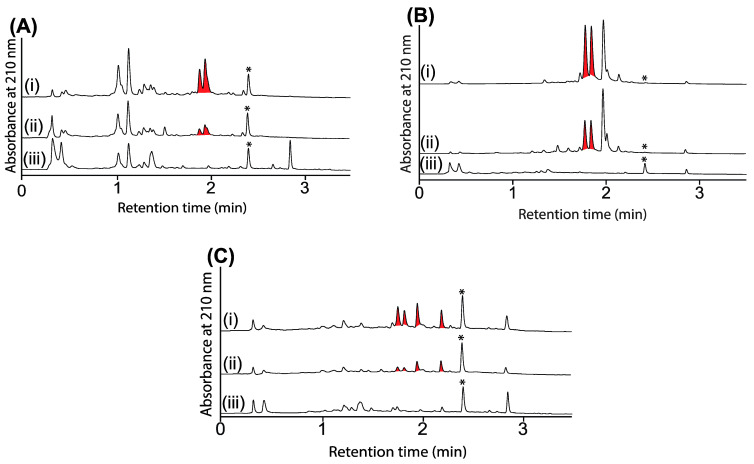
UPLC-DAD (210 nm) chromatograms of cultivations of (**A**) CMB-M0139, (**B**) CMB-GO014 and (**C**) S4S-00182 (i) with and (ii) without *N*-aminoanthranilic acid (30 μM) and (iii) media only, with all transcriptionally activated metabolites highlighted in red, and where * is an internal calibrant.

To test the hypothesis that **11** may exert an ecological survival benefit as a natural transcriptional regulator, we used our 24-well plate microbioreactor (MATRIX) strategy to cultivate a panel of ×11 bacterial (Table S5) and ×11 fungal (Table S6) isolates (selected from our inhouse libraries) in the absence and presence of **11** at a noncytotoxic concentration (30 μM in 1% DMSO in culture broth). Subsequent UPLC-DAD and UPLC-QTOF-MS/MS (GNPS) analysis of in situ EtOAc extracts confirmed that two fungal isolates experienced transcriptional activation. For example, the fungus CMB-W003 recovered from a mud dauber wasp, and currently the subject of inhouse (unpublished) investigation under multiple cultivation conditions, underwent transcriptional activation when exposed to **11** to produce bacteriostatic roquefortine C [13,14,15] and the biosynthetically related cytotoxic tubulin polymerisation inhibitor oxaline (Figure 5A) [16]. Likewise, the fungus *Penicillium* sp. CMB-MD22 isolated from a mud dauber wasp nest and previously studied extensively under multiple culture conditions and reported as a source of new selectively antifungal neobulgarones, [17] underwent transcriptional activation when exposed to **11** to produce silent chemical defenses in the form of potentially new neobulgarone analogues (Figure 5B). Furthermore, exposure to **11** also resulted in transcriptional enhancement and increased production yields of an array of natural products in the bacteria CMB-M0139 and fungi CMB-GO014 and S4S-00182 (Figure 6). It is worthwhile noting that in each of the five cases outlined above, our MATRIX cultivation profiling strategy (×33 different culture conditions per microbe) did not lead to the activation/enhancement observed after supplementation with *N*-aminoanthranilic acid. While preliminary in scope, these experiments demonstrate that **11** changed the transcriptional state of natural product biosynthesis in 4/10 fungi and in 1/10 bacteria tested. Further investigation into the potential application of **11** as a culture media additive to enable access to new microbial chemical space seems warranted, particularly across a broader panel of taxonomically diverse isolates.

In summary, our successful total synthesis and structure revision of penipacids A–E (**6**–**10**) provided a compelling case that some if not all of the penipacids were Schiff base adduct artifacts generated through conjugation of the highly reactive cryptic natural product *N*-aminoanthranilic acid (**11**) with diacetone alcohol, diacetone alcohol methyl ether, pyruvic acid, methyl pyruvate or furfural. The artifact status of penipacids A–B (**6**–**8**) was particularly compelling given excessive exposure to acetone and methanol under acidic conditions during fractionation and handling. We go on to demonstrate that while **11** exhibited no obvious cytotoxicity against a panel of bacterial, fungal and mammalian cells, it did act as a natural transcriptional regulator of silent biosynthetic genes encoding for other natural product chemical defenses. Collectively, these observations highlight the value of informed questioning of the likely biosynthesis of new natural products, and where needed, the willingness to question assigned structures. We also highlight the value of recognizing and documenting the distinction between natural products and artifacts and the potential to using artifacts as markers for cryptic and highly reactive natural products. Finally, we provide preliminary evidence that the cryptic and highly reactive putative natural product *N*-aminoanthranilic acid (**11**) is a useful molecular tool as a chemical additive to microbial cultivations capable of in situ activation of silent (or low yield) biosynthetic gene clusters.

## 3. Materials and Methods

### 3.1. General Experimental Procedures

Chemicals were purchased from Sigma-Aldrich or Merck unless otherwise specified. Analytical-grade solvents were used for solvent extractions. Solvents used for HPLC, UPLC and HPLC-MS purposes were of HPLC grade supplied by Labscan or Sigma-Aldrich, Brisbane, Australia and filtered/degassed through 0.45 μm polytetrafluoroethylene (PTFE) membrane prior to use. Deuterated solvents were purchased from Cambridge Isotopes (Tewksbury, MA, USA). Semipreparative HPLC was performed using Agilent 1100 series HPLC instruments with corresponding detectors, fraction collectors and software inclusively. Liquid chromatography-diode array-mass spectrometry (HPLC-DAD-MS) data were acquired on an Agilent 1260 Infinity II separation module equipped with an Agilent single quad MSD mass detector and diode array multiple wavelength detector using Agilent Zorbax SB-C_8_ column (2.1 × 150 mm, 3.5 mm) running 0.8 mL/min gradient elution from 90% H_2_O/MeCN to 100% MeCN (with constant 0.05% formic acid modifier) over 6.5 min. Nuclear magnetic resonance (NMR) spectra were acquired on a Bruker Avance 600 MHz spectrometer with either a 5 mm PASEL 1H/D-13C Z-gradient probe or 5 mm CPTCI 1H/19F-13C/15N/DZ-gradient cryoprobe, controlled by TopSpin 2.1 software, Bruker, Massachusetts, USA. In all cases, spectra were acquired at 25 °C in DMSO-*d*_6_ with reference to residual ^1^H or ^13^C signals (δ_H_ 2.50 and δ_C_ 39.51); CDCl_3_ with reference to residual ^1^H or ^13^C signals (δ_H_ 7.24 and δ_C_ 77.23); and methanol-*d*_4_ with reference to residual ^1^H or ^13^C signals (δ_H_ 3.31 and δ_C_ 49.15) in the deuterated solvent. High-resolution ESIMS spectra were obtained on a Bruker micrOTOF mass spectrometer by direct injection in MeOH at 3 μL/min using sodium formate clusters as an internal calibrant.

### 3.2. Synthesis of Penipacids

*Synthesis of N-aminoanthranilic acid (**11**).* Following a synthetic procedure adopted from Dang et al. [2], anthranilic acid (274 mg, 2.00 mmol) in 6M HCl (4.80 mL) cooled to 0 °C was stirred for 10 min, followed by dropwise addition of a solution of NaNO_2_ (152 mg, 2.30 mmol) in water (0.8 mL). The reaction mixture was stirred at 0 °C for 1 h, and then a solution of SnCl_2_.2H_2_O (903 mg, 4.00 mmol) in 6M HCl (1.20 mL) was added, and the mixture was stirred at room temperature for 1 h, followed by standing for 2 h at 4 °C. The resulting precipitate was filtered under vacuum and washed 3 times with ice cold water to yield *N*-aminoanthranilic acid (**11**) (173 mg, 57% yield, *R_f_* 0.42 in 10% MeOH/CH_2_Cl_2_). HRESI(+)MS calculated for C_7_H_9_N_2_O_2_ 153.0659, found 153.0657. ^1^H NMR (600 MHz, DMSO-*d*_6_) δ_H_ 10.50 (1H, br s), 9.08 (1H, br s), 7.93 (1H, d, 7.7), 7.58 (1H, dd, 8.2, 1.1), 7.12 (1H, d, 8.2), 6.97 (1H, dd, 8.2, 7.7); ^13^C NMR (150 MHz, DMSO-*d*_6_) δ_C_ 169.3, 148.3, 134.7, 131.8, 120.1, 114.1, 113.9. (Figures S4–S7).

*Synthesis of penipacid A (**6**). N*-aminoanthranilic acid (**11**) (45 mg, 0.30 mmol) in diacetone alcohol (37.3 μL, 0.30 mmol, 34.8 mg) was stirred at room temperature for 5 min, with thin layer chromatography and HPLC-MS analysis revealing quantitative conversion. Flash silica column chromatography (0–10% MeOH/CH_2_Cl_2_) yielded a pure sample of penipacid A (**6**) as a yellow oil. HRESI(+)MS calculated for C_13_H_18_N_2_O_3_Na 273.1210, found 273.1212. ^1^H and ^13^C NMR (600 MHz, CDCl_3_) data were identical with those of the reported natural product penipacid A (**1**) [1] (Table S1 and Figures S8–S11).

*Synthesis of penipacid B (**7**). N*-aminoanthranilic acid (**11**) (45 mg, 0.30 mmol) in the methyl ether of diacetone alcohol (43.8 μL, 0.30 mmol, 39 mg) was stirred at room temperature for 5 min, with thin layer chromatography and HPLC-MS analysis revealing quantitative conversion. Semipreparative reversed-phase HPLC (column Zorbax C_8_ Eclipse, 5 μm, 9.4 × 250 mm column, 20 min 3 mL/min isocratic elution as 65% MeCN/H_2_O with a 0.1% TFA/MeCN modifier) yielded a pure sample of penipacid B (**7**) as a yellow oil. HRESI(+)MS calculated for C_14_H_20_N_2_O_3_Na 287.1366, found 287.1379. ^1^H and ^13^C NMR (600 MHz, methanol-*d*_4_) data were identical with those of the reported natural product penipacid B (**2**) [1] (Table S2 and Figures S12–S15).

*Synthesis of penipacid C (**8**). N*-aminoanthranilic acid (**11**) (45 mg, 0.30 mmol) and pyruvic acid (26 mg, 0.20 mmol) in MeOH (5 mL) was stirred at room temperature for 10 min, with thin layer chromatography and HPLC-MS analysis revealing quantitative conversion. Semipreparative reversed-phase HPLC (column Zorbax C_8_ Eclipse, 5 μm, 9.4 × 250 mm column, 30 min 3 mL/min gradient elution from H_2_O to 35% H_2_O/MeCN with an isocratic 0.1% TFA/MeCN modifier) yielded a pure sample of penipacid C (**8**) as a yellow oil. HRESI(+)MS calculated for C_10_H_10_N_2_O_4_Na 245.0533, found 245.0539. ^1^H and ^13^C NMR (600 MHz, DMSO-*d*_6_) data were identical with those of the reported natural product penipacid C (**3**) [1] (Table S3 and Figures S16–S19).

*Synthesis of penipacid D (**9**). N*-aminoanthranilic acid (**11**) (45 mg, 0.3 mmol) in methyl pyruvate (27 μL, 0.30 mmol, 30.6 mg) was stirred at room temperature for 10 min, with thin layer chromatography and HPLC-MS analysis revealing quantitative conversion. Semipreparative reversed-phase HPLC (column Zorbax C_8_ Eclipse, 5 μm, 9.4 × 250 mm column, 30 min 3 mL/min gradient elution from H_2_O to 40% H_2_O/MeCN with an isocratic 0.1% TFA/MeCN modifier) yielded a pure sample of penipacid D (**9**) as a yellow oil. HRESI(+)MS calculated for C_11_H_12_N_2_O_4_Na 259.0689, found 259.0696. ^1^H and ^13^C NMR (600 MHz, methanol-*d*_4_) data were identical with those of the reported natural product penipacid D (**4**) [1] (Table S4 and Figures S20–S23).

*Synthesis of penipacid E (**10**). N*-aminoanthranilic acid (**11**) (45 mg, 0.3 mmol, 1 eq.) in furfural (24.8 μL, 0.30 mmol, 28.8 mg) was stirred at room temperature for 10 min, with thin layer chromatography and HPLC-MS analysis revealing partial conversion. Semipreparative reversed-phase HPLC (column Zorbax Phenyl, 5 μm, 9.4 × 250 mm column, 30 min 3 mL/min gradient elution from H_2_O to 35% H_2_O/MeCN with an isocratic 0.1% TFA/MeCN modifier) yielded a pure (trace) sample of penipacid E (**10**) as a yellow oil. HRESI(+)MS calculated for C_12_H_10_N_2_O_3_Na 253.0584, found 253.0579 (Figures S24 and S25).

### 3.3. Antibacterial Assay

The bacterium to be tested was streaked onto a tryptic soy agar plate and was incubated at 37 °C for 24 h. One colony was then transferred to fresh tryptic soy broth (5 mL), and the cell density was adjusted to 10^4^–10^5^ CFU/mL. N-amino anthranilic acid was dissolved in DMSO and diluted with H_2_O to give 600 μM stock solution (20% DMSO), which was serially diluted with 20% DMSO to give concentrations from 600 μM to 0.2 μM in 20% DMSO. An aliquot (10 μL) of each dilution was transferred to a 96-well microtiter plate, and freshly prepared microbial broth (190 μL) was added to each well to give final concentrations of 30–0.01 μM in 1% DMSO. The plates were incubated at 37 °C for 24 h, and the optical density (OD) of each well was measured spectrophotometrically at 600 nm using POLARstar Omega plate (BMG LABTECH, Offenburg, Germany). Each test compound was screened against the Gram-negative bacterium *Escherichia coli* ATCC11775 and the Gram-positive bacteria *Staphylococcus aureus* ATCC25923 and *Bacillus subtilis* ATCC6633. A mixture of rifampicin and ampicillin was used as a positive control (30 μM in 20% DMSO). The IC_50_ value was calculated as the concentration of the compound or antibiotic required for 50% inhibition of the bacterial cells using Prism 8.0 (GraphPad Software Inc., La Jolla, CA, USA) (Figure S26).

### 3.4. Antifungal Assay

The fungus *Candida albicans* ATCC 10231 was streaked onto a tryptic soy agar plate and was incubated at 37 °C for 48 h. One colony was then transferred to fresh tryptic soy broth (5 mL), and the cell density was adjusted to 10^4^–10^5^ CFU/mL. N-amino anthranilic acid was dissolved in DMSO and diluted with H_2_O to give a 600 μM stock solution (20% DMSO), which was serially diluted with 20% DMSO to give concentrations from 600 μM to 0.2 μM in 20% DMSO. An aliquot (10 μL) of each dilution was transferred to a 96-well microtiter plate, and freshly prepared fungal broth (190 μL) was added to each well to give final concentrations of 30–0.01 μM in 1% DMSO. The plates were incubated at 37 °C for 24 h, and the optical density (OD) of each well was measured spectrophotometrically at 600 nm using POLARstar Omega plate (BMG LABTECH, Offenburg, Germany). Amphotericin B was used as a positive control (30 μg/mL in 20% DMSO). Where relevant, IC_50_ value were calculated as the concentration of the compound or antifungal drug required for 50% inhibition of the fungal cells using Prism 8.0 (GraphPad Software Inc., La Jolla, CA, USA) (Figure S26).

### 3.5. Cytotoxicity (MTT) Assay

The MTT assay was slightly modified from that previously described [3]. Adherent SW620 (susceptible human colorectal carcinoma) and NCIH-460 (human lung carcinoma) were cultured in Roswell Park Memorial Institute (RPMI) 1640 medium. All cells were cultured as adherent monolayers in flasks supplemented with 10% foetal bovine serum, L–glutamine (2 mM), penicillin (100 unit/mL) and streptomycin (100 μg/mL) in a humidified 37 °C incubator supplied with 5% CO_2_. Briefly, cells were harvested with trypsin and dispensed into 96-well microtiter assay plates at 8000 cells/well after which they were incubated for 18 h at 37 °C with 5% CO_2_ (to allow cells to attach as adherent monolayers). N-amino anthranilic acid was dissolved in 20% DMSO in PBS (*v*/*v*), and aliquots (10 μL) were applied to cells over a series of final concentrations ranging from 10 nM to 30 μM. After 48 h incubation at 37 °C with 5% CO_2,_ an aliquot (10 μL) of 3-(4,5-dimethylthiazol-2-yl)-2,5-diphenyltetrazolium bromide (MTT) in phosphate buffered saline (PBS, 5 mg/mL) was added to each well (final concentration 0.5 mg/mL), and microtiter plates were incubated for a further 4 h at 37 °C with 5% CO_2_. After final incubation, the medium was aspirated, and precipitated formazan crystals were dissolved in DMSO (100 μL/well). The absorbance of each well was measured at 600 nm with a POLARstar Omega plate (BMG LABTECH, Offenburg, Germany). Where relevant, IC_50_ values were calculated using Prism 8.0, as the concentration of analyte required for 50% inhibition of cancer cell growth (compared to negative controls). Negative control was 1% aqueous DMSO, while positive control was doxorubicin (final concentrations from 10 nM to 30 μM). All experiments were performed in duplicate from two independent cultures (Figure S27).

### 3.6. Activation Studies

Microbioreactor cultures of the Australian Collection of Microbes (ACM), Centre for Microbial Biodiscovery (CMB) and Soils for Science (S4S) strains (Table S6) were treated with *N*-amino anthranilic acid (15 μL, 30 μM in 1% DMSO) on day 0 in broth medium (1.5 mL). Positive controls were inoculated with seed culture (15 μL) of the strain, while negative controls contained medium only. Both positive and negative controls were treated with 1% DMSO/H_2_O per well. All microbioreactor plates were incubated at 27 °C for 7 days at 190 rpm after which the broth was extracted in situ with EtOAc (2 mL), and the decanted organic layer was dried under N_2_. The resulting crude EtOAc extracts were resuspended in MeOH (100 μL) and analyzed using UPLC-DAD and UPLC QTOF.

## Supplementary Materials

The following supporting information can be downloaded at: https://www.mdpi.com/article/10.3390/md20060339/s1, Figures S1–S3: synthesis of anthranilic acid; Figures S4–S7: synthesis of N-amino anthranilic acid (**11**); Figures S8–S25: Synthesis of penipacids A-E (**6**-**10**) and the tabulated 1D and 2D NMR data, annotated 1D NMR and selected 2D NMR spectra; Figure S26: growth inhibitory activity of N-aminoanthranilic acid; Figure S27: cytotoxicity of N-aminoanthranilic acid against SW620 and NCIH-460. Table S1: 1D NMR data for penipacid A (**6**); Table S2: 1D NMR data for penipacid B (**6**); Table S3: 1D NMR data for penipacid C (**8**); Table S4: 1D NMR data for penipacid D (**9**); Table S5: list of bacterial strains for the activation trials; Table S6: List of fungal strains for the activation trials.
