# Supplementary material for: Structure Revision of Penipacids A–E Reveals a Putative New Cryptic Natural Product, N-aminoanthranilic Acid, with Potential as a Transcriptional Regulator of Silent Secondary Metabolism
Source: Mar Drugs. 2022 May 24;20(6):339. doi: 10.3390/md20060339 (PMC9225364; doi:10.3390/md20060339)
Supplement: Supplementary file 1 [file marinedrugs-20-00339-s001.zip › marinedrugs-1719543-supplementary.pdf]

Supplementary Material

Structure revision of penipacids A–E reveals a putative new cryptic natural product, *N*-aminoanthranilic acid, with potential as a transcriptional regulator of silent secondary metabolism.

Zeinab G. Khalil†, Sarani Kankanamge† and Robert J. Capon *

Institute for Molecular Bioscience, The University of Queensland, St Lucia, QLD, Australia; z.khalil@uq.edu.au (Z.G.K)

Institute for Molecular Bioscience, The University of Queensland, St Lucia, QLD, Australia; s.kankanamge@imb.uq.edu.au (S.D.R)

†These authors contributed equally

* Correspondence: r.capon@uq.edu.au (R.J.C.); Tel.: +61 7 3346 2979

Table of contents

1 General experimental details 4
2 Synthesis of anthranilic acid 5
3 Synthesis of *N*-aminoanthranilic acid (**11**) 8
4 Synthesis of penipacid A (**6**) 11
5 Synthesis of penipacid B (**7**) 14
6 Synthesis of penipacid C (**8**) 17
7 Synthesis of penipacid D (**9**) 20
8 Synthesis of penipacid E (**10**) 23
9 Biological assays 25
9.1 Antibacterial and antifungal assays 25
9.2 Cytotoxicity (MTT) assay 25
10 References 27

List of Figures

Figure S1. HPLC-DAD-MS chromatogram for the reaction mixture of anthranilic acid. 6
Figure S2. ^{1}H NMR (600 MHz, DMSO-d_6) spectrum of anthranilic acid 7
Figure S3. ^{13}C (150 MHz, DMSO-d_6) spectrum of anthranilic acid 7
Figure S4. HPLC-DAD-MS chromatogram for the reaction mixture of *N*-aminoanthranilic acid 8
Figure S5. ^{1}H NMR (600 MHz, DMSO-d_6) spectrum of *N*-aminoanthranilic acid (**11**) 9
Figure S6. ^{13}C (150 MHz, DMSO-d_6) spectrum of *N*-aminoanthranilic acid (**11**) 9
Figure S7. HRMS for *N*-aminoanthranilic acid (**11**) 10
Figure S8. HPLC-DAD chromatogram for the purified penipacid A 11
Figure S9. ^{1}H NMR (600 MHz, $CDCl_3$) for penicpacid A (**6**) 12
Figure S10. ^{13}C (150 MHz, $CDCl_3$) spectrum for penipacid A (**6**) 12
Figure S11. HRMS for penipacid A (**6**) 13
Figure S12. HPLC-DAD (210 nm) chromatogram for the purified penipacid B. 14
Figure S13. ^{1}H NMR (600 MHz, methanol-d_4) for penicpacid B (**7**) 15
Figure S14. ^{13}C (150 MHz, methanol-d_4) spectrum for penipacid B (**7**) 15
Figure S15. HRMS for penipacid B (**7**) 16
Figure S16. HPLC-DAD chromatogram for the purified penipacid C. 17
Figure S17. ^{1}H NMR (600 MHz, DMSO-d_6) for penipacid C (**8**) 18
Figure S18. ^{13}C (150 MHz, DMSO-d_6) for penipacid C (**8**) 18
Figure S19. HRMS for penipacid C (**8**) 19
Figure S20. HPLC-DAD chromatogram for the purified penipacid D 20
Figure S21. ^{1}H NMR (600 MHz, methanol-d_4) for penipacid D (**9**) 21
Figure S22. ^{13}C (150 MHz, methanol-d_4) for penipacid D (**9**) 21
Figure S23. HRMS for penipacid D (**9**) 22
Figure S24. HPLC-DAD chromatogram for the reaction mixture of penipacid E 23
Figure S25. HRMS for penipacid E (**10**) 24
Figure S26. Growth inhibitory activity of *N*-aminoanthranilic acid 25
Figure S27. Cytotoxicity of *N*-aminoanthranilic acid against SW620 and NCIH-460. 25

List of Tables

Table S1. 1D NMR (600 MHz, $CDCl_3$) data for penipacid A (**6**) 11
Table S2. 1D NMR (600 MHz, methanol-d_4) data for penipacid B (**7**) 14
Table S3. 1D NMR (600 MHz, DMSO-d_6) data for penipacid C (**8**).................................... 17
Table S4. 1D NMR (600 MHz, methanol-d_4) data for penipacid D (**9**) 20
Table S5. List of bacterial strains for the activation trials... 26
Table S6. List of fungal strains for the activation trials 26

List of Schemes

Scheme S1.Synthesis of anthranilic acid 5
Scheme S2. Synthesis of *N*-aminoanthranilic acid from anthranilic acid. 8
Scheme S3. Synthesis of penipacid A (**6**) from *N*-aminoanthranilic acid 11
Scheme S4. Synthesis of penipacid B (**7**) from *N*-aminoanthranilic acid 14
Scheme S5. Synthesis of penipacid C (**8**) from *N*-aminoanthranilic acid 17
Scheme S6. Synthesis of penipacid D (**9**) from *N*-aminoanthranilic acid 20
Scheme S7. Synthesis of penipacid E (**10**) from *N*-aminoanthranilic acid 23

1 General experimental details

Chemicals were purchased from Sigma-Aldrich or Merck unless otherwise specified. Analytical-grade solvents were used for solvent extractions. Solvents used for HPLC, UPLC and HPLC-MS purposes were of HPLC grade supplied by Labscan or Sigma-Aldrich and filtered/degassed through 0.45 μm polytetrafluoroethylene (PTFE) membrane prior to use. Deuterated solvents were purchased from Cambridge Isotopes (Tewksbury, MA, USA).

Semi-preparative HPLC was performed using Agilent 1100 series HPLC instruments with corresponding detectors, fraction collectors and software inclusively. Liquid chromatography-diode array-mass spectrometry (HPLC-DAD-MS) data was acquired on an Agilent 1260 Infinity II separation module equipped with an Agilent single quad MSD mass detector and diode array multiple wavelength detector using Agilent Zorbax SB-C_8 column (2.1 × 150 mm, 3.5 mm) running 0.8 mL/min gradient elution from 90% H_2O/MeCN to 100% MeCN (with constant 0.05% formic acid modifier) over 6.5 min. UPLC system equipped with diode array multiple wavelength detector (Zorbax C_8 RRHD 1.8 μm column, 50 × 2.1 mm, eluting with 0.417 mL/min 90% H_2O/MeCN to 100% MeCN (with isocratic 0.01% TFA modifier) over 2.50 min). UHPLC-QTOF analysis was performed on UHPLC-QTOF instrument comprising an Agilent 1290 Infinity II UHPLC (Zorbax C_8 RRHD 1.8 μm column, 50 × 2.1 mm, eluting with 0.417 mL/min of isocratic 90% H_2O/MeCN to 100% MeCN over 2.50 min (with isocratic 0.1% formic acid modifier) coupled to an Agilent 6545 Q-TOF. MS/MS analysis was performed on the same instrument for ions detected in the full scan at an intensity above 1000 counts at 10 scans/s, with an isolation width of 4 ~m/z using a fixed collision energy and a maximum of 3 selected precursors per cycle. Nuclear magnetic resonance (NMR) spectra were acquired on a Bruker Avance 600 MHz spectrometer with either a 5 mm PASEL 1H/D-13C Z-Gradient probe or 5 mm CPTCI 1H/19F-13C/15N/DZ-Gradient cryoprobe, controlled by TopSpin 2.1 software. In all cases spectra were acquired at 25 °C in DMSO-d_6 with referencing to residual ^1H or ^{13}C signals (δ_H 2.50 and δ_C 39.51 ppm); $CDCl_3$ with referencing to residual ^1H or ^{13}C signals (δ_H 7.24 and δ_C 77.23 ppm) and MeOH-d_4 with referencing to residual ^1H or ^{13}C signals (δ_H 3.31 and δ_C 49.15 ppm) in the deuterated solvent. High-resolution ESIMS spectra were obtained on a Bruker micrOTOF mass spectrometer by direct injection in MeOH at 3 μL/min using sodium formate clusters as an internal calibrant.

2 Synthesis of anthranilic acid

Anthranilic acid was prepared by reacting Na-phthalimide with sodium hypochlorite followed by neutralization with conc. HCl. Briefly, solid NaOH (9 g) was dissolved in water (100 mL) and the reaction mixture was cooled down to 10 °C, stirred for 5 min. Phthalimide (15 g) was added to the solution and stirred for 10 min at 10 °C. Following that sodium hypochlorite (7 g) was added to the mixture and further stirred for another 15 min, after which the reaction mixture was heated to 50 °C for 15 min and then cooled down to 30 °C. Conc. HCl (30 mL) was added to neutralize the pH followed by the addition of glacial acetic acid (30 mL) dropwise. The formed crystals were obtained by filtration and washed with cold water to afford anthranilic acid in a quantitative yield as grey powder. LRMS 138 $[M+H]^+$ and 136 $[M+H]^-$. ^1H NMR (600 MHz, DMSO-d_6) δ_H 7.68 (1H, dd, *7.9, 1.5*), 7.19 (1H, dd, *8.4, 1.5*), 6.72 (1H, dd, *8.4, 0.7*), 6.48 (1H, dd, *8.4, 1.0*); ^{13}C NMR (150 MHz, DMSO-d_6) δ_C 170.2, 151.8, 133.8, 131.6, 116.7, 114.9, 110.8.

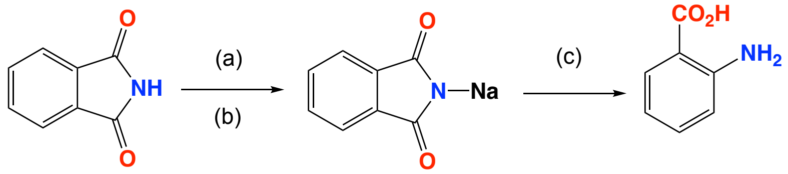

Scheme S1. Synthesis of anthranilic acid using (a) NaOH for 10 amin at 10 °C; (b) NaClO for 15 min at 50 °C then reaction mixture was cooled to 30 °C and (c) conc. HCl for 10 min at 30 °C followed by the addition of glacial acetic acid for 5 min

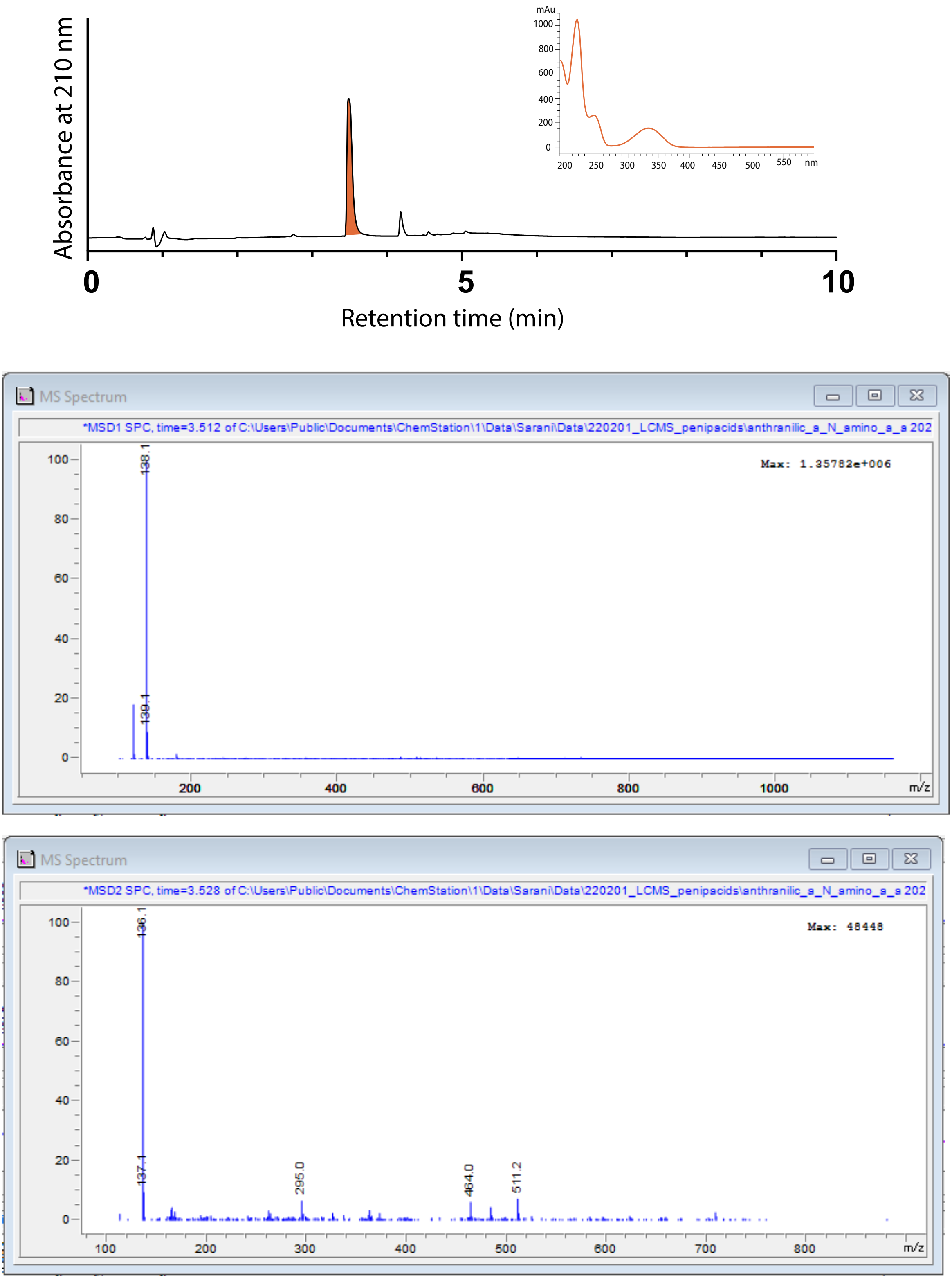

Figure S1. HPLC-DAD-MS (210 nm) chromatogram for the reaction mixture of anthranilic acid (highlighted peak, retention time 4.17 min) and UV vis spectrum inset using Agilent Zorbax SB-C_8 column (2.1 × 150 mm, 3.5 mm) running 0.8 mL/min gradient elution from 90% H_2O/MeCN to 100% MeCN (with constant 0.05% formic acid modifier) over 6.5 min.

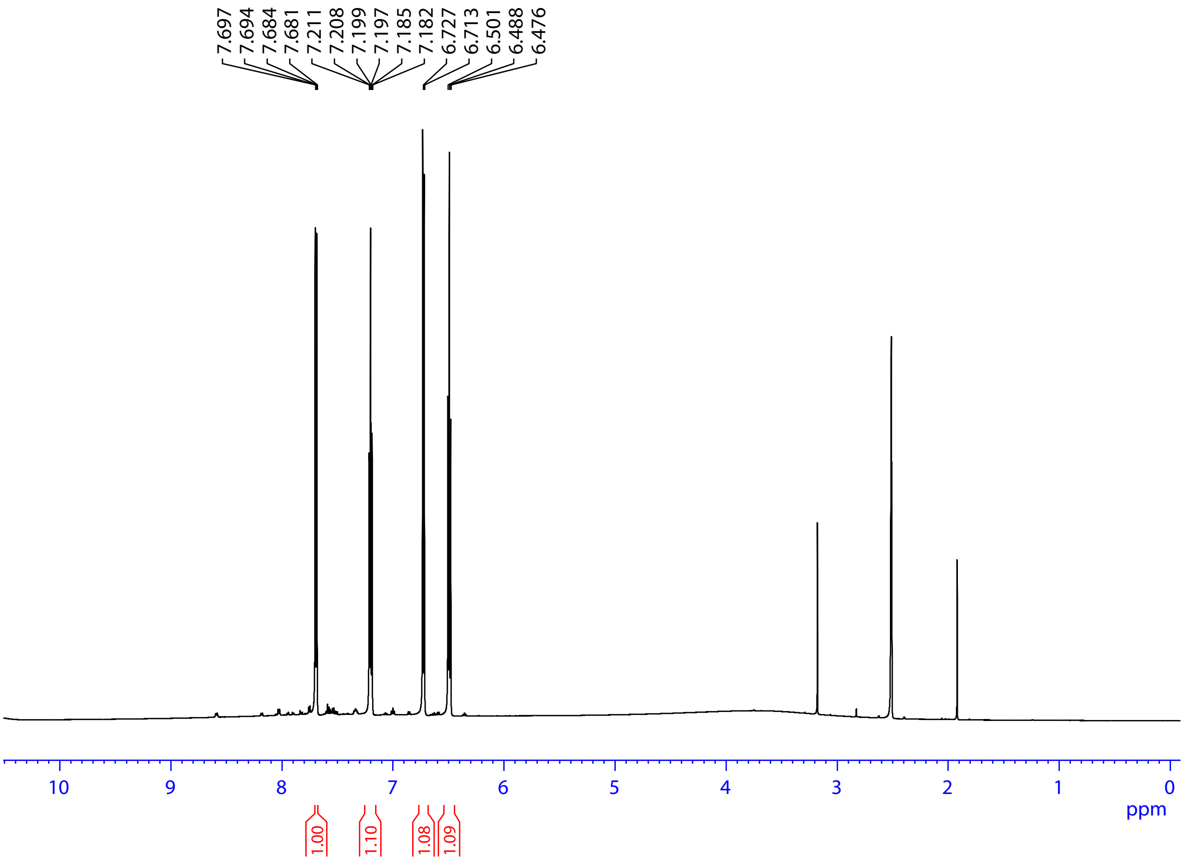

Figure S2. ^{1}H NMR (600 MHz, DMSO-d_6) spectrum of anthranilic acid

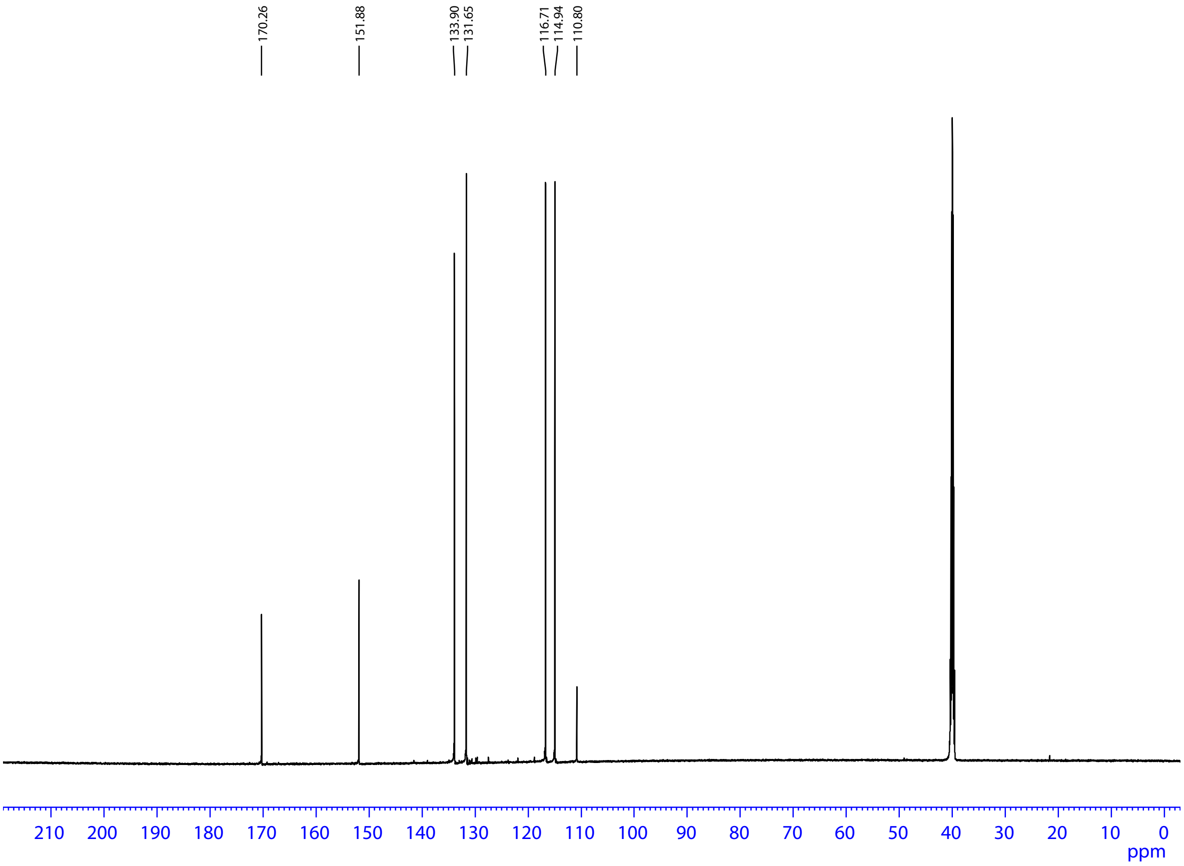

Figure S3. ^{13}C (150 MHz, DMSO-d_6) spectrum of anthranilic acid

3 Synthesis of *N*-aminoanthranilic acid (11)

The synthetic procedure was adopted from the method by Dang et al.[2] Anthranilic acid (274 mg, 2 mmol) was dissolved in 6M HCl (4.8 mL) and the reaction mixture was cooled down to 0 °C in ice and stirred for 10 min, followed by dropwise addition of a solution of $NaNO_2$ (152 mg, 2.3 mmol) in water (0.8 mL). The reaction mixture was stirred for 1 h at 0 °C, then the solution of $SnCl_2.2H_2O$ (903 mg, 4 mmol) in 6M HCl (1.2 mL) was added. The ice bath was removed, and the reaction mixture was stirred for another 1 h at room temperature and then set-aside in the cold room for 2 h at 4 °C. The formed precipitate was filtered under vacuum, washed 3 times with ice cold water to yield *N*-aminoanthranilic acid (173 mg, 57% yield, R_f 0.42 in 10% MeOH/DCM, the spot was visualized by UV at 254 nm). HRESI(+)MS calculated for $C_7H_9N_2O_2$ 153.0659, found 153.0657. ^{1}H NMR (600 MHz, DMSO-d_6) δ_H 10.50 (1H, br s), 9.08 (1H, br s), 7.93 (1H, d, *7.7*), 7.58 (1H, dd, *8.2, 1.1*), 7.12 (1H, d, *8.2*), 6.97 (1H, dd, *8.2, 7.7*); ^{13}C NMR (150 MHz, DMSO-d_6) δ_C 169.3, 148.3, 134.7, 131.8, 120.1, 114.1, 113.9.

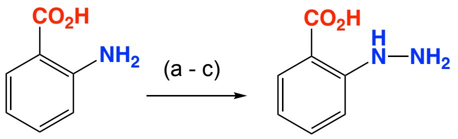

Scheme S2. Synthesis of *N*-aminoanthranilic acid from anthranilic acid. (a) 6M HCl, (b) $NaNO_2$ and (c) $SnCl_2.2H_2O$

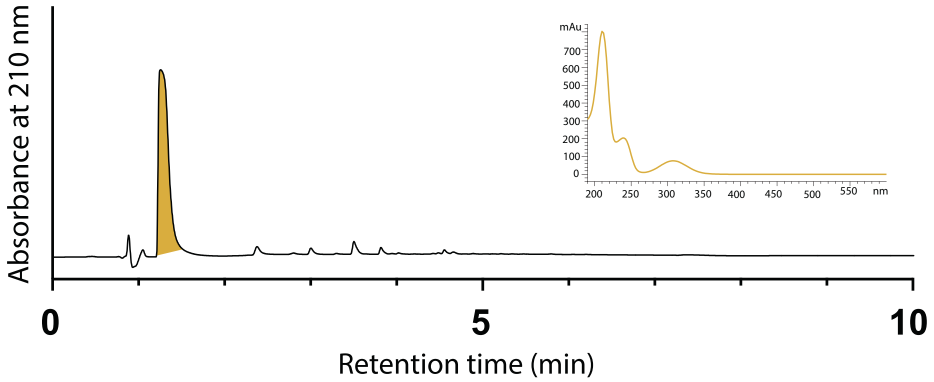

Figure S4. HPLC-DAD-MS (210 nm) chromatogram for the reaction mixture of *N*-aminoanthranilic acid (highlighted peak, retention time 4.17 min) and UV vis spectrum inset using Agilent Zorbax SB-C_8 column (2.1 × 150 mm, 3.5 mm) running 0.8 mL/min gradient elution from 90% H_2O/MeCN to 100% MeCN (with constant 0.05% formic acid modifier) over 6.5 min

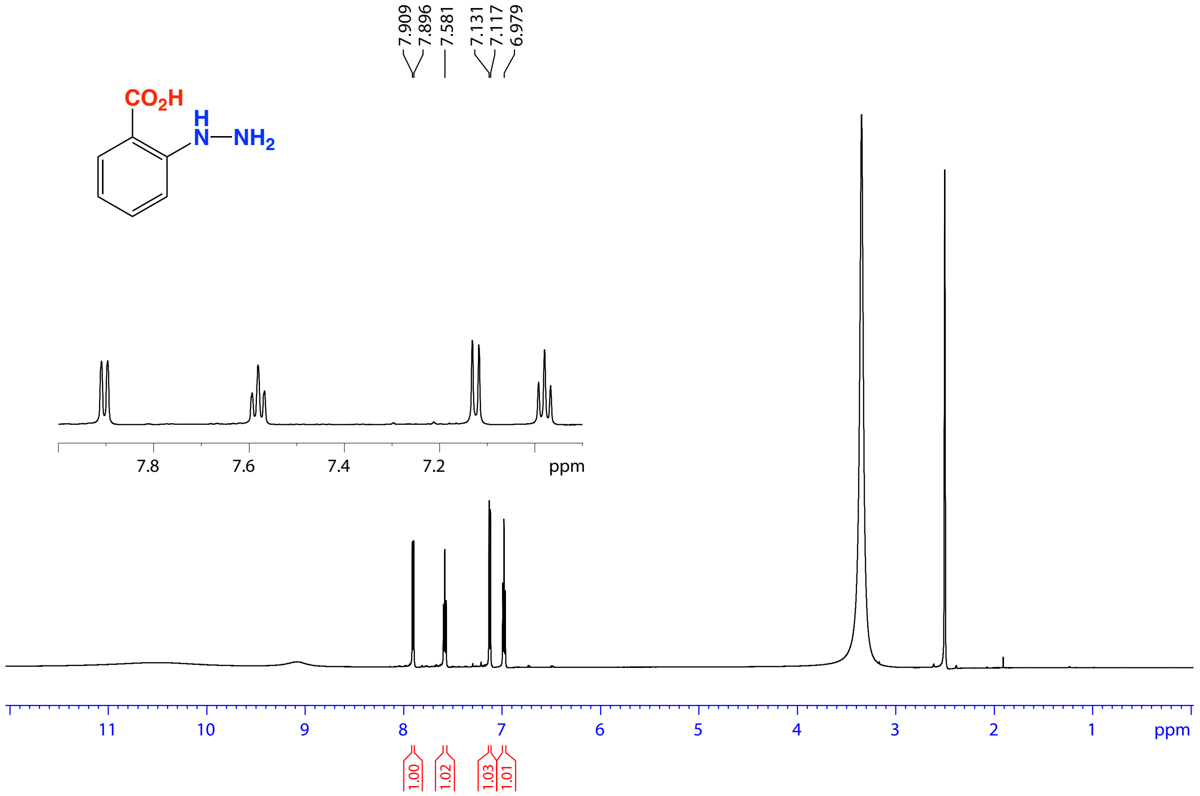

Figure S5. ^{1}H NMR (600 MHz, DMSO-d_6) spectrum of *N*-aminoanthranilic acid (**11**)

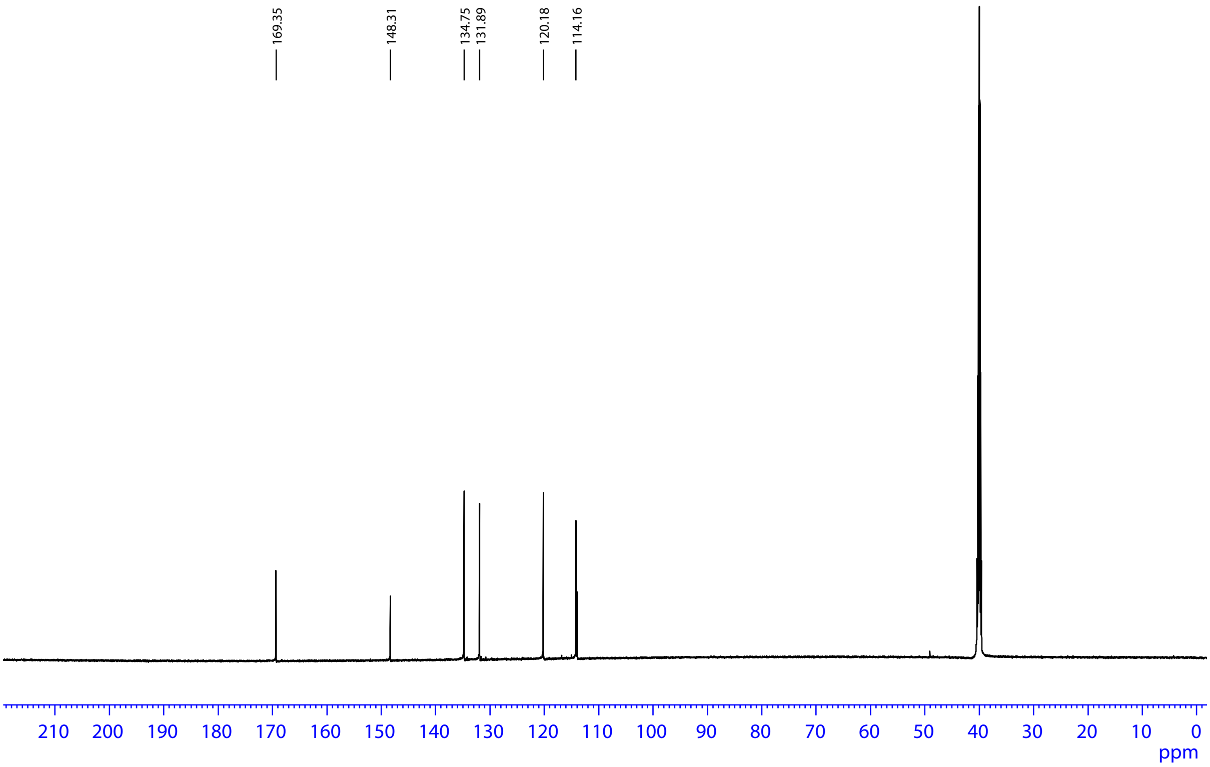

Figure S6. ^{13}C (150 MHz, DMSO-d_6) spectrum of *N*-aminoanthranilic acid (**11**)

Mass Spectrum Molecular Formula Report

Analysis Info

Analysis Name	D:\Data\s.kankanamge\N_aminoanthranilic acid_2.d	Acquisition Date	6/23/2021 3:46:59 PM
Method	tune-low_AP.m	Operator	a.salim
Sample Name	N_aminoanthranilic acid_2	Instrument / Ser#	micrOTOF 213750.00232
Comment			

Acquisition Parameter

Source Type	ESI	Ion Polarity	Positive	Set Nebulizer	0.8 Bar
Focus	Not active			Set Dry Heater	180 °C
Scan Begin	100 m/z	Set Capillary	4500 V	Set Dry Gas	5.0 l/min
Scan End	1000 m/z	Set End Plate Offset	-500 V	Set Divert Valve	Source

Generate Molecular Formula Parameter

Formula, min.		
Formula, max.		
Measured m/z	Tolerance	Charge
Check Valence	Minimum	Maximum
Nirogen Rule	Electron Configuration	
Filter H/C Ratio	Minimum	Maximum
Estimate Carbon		

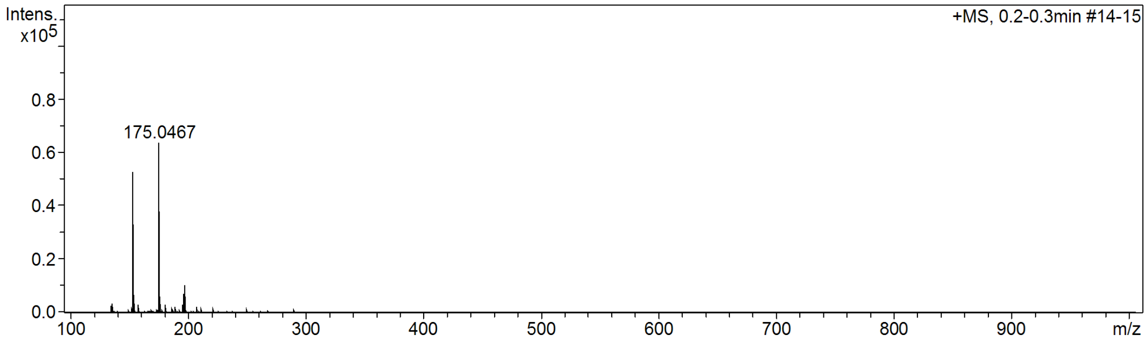

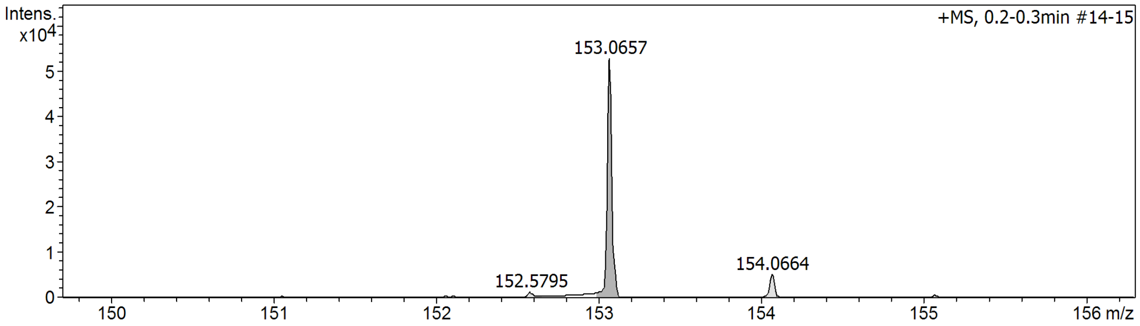

Meas. m/z	#	Ion Formula	m/z	err [ppm]	mSigma	# Sigma	Score	rdb	e¯ Conf	N-Rule
153.0657	1	C7H9N2O2	153.0659	-1.3	8.0	1	100.00	4.5	even	ok
175.0467	1	C7H8N2NaO2	175.0478	6.1	1.5	1	100.00	4.5	even	ok

Figure S7. HRMS for *N*-aminoanthranilic acid (**11**)

4 Synthesis of penipacid A (6)

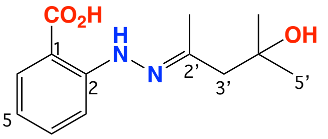

Penipacid A was synthesized by reacting *N*-aminoanthranilic acid (45 mg, 0.3 mmol.) with diacetone alcohol (0.3 mmol, 34.8 mg, 37.3 µL). The reaction mixture was stirred for 5 min at room temperature. The crude product was purified using flash column chromatography (0-10% MeOH/DCM) to yield penipacid A in a quantitative yield as yellow oil; R_f: 0.52 (10% MeOH/DCM and the spot was visualized by UV at 254 nm). HRESI(+)MS calculated for $C_{13}H_{18}N_2O_3Na$ 273.1210, found 273.1212.

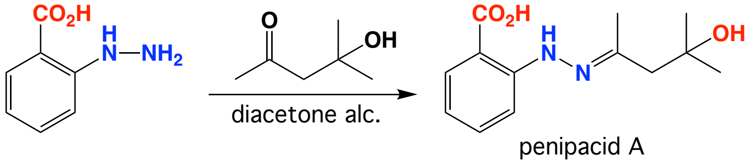

Scheme S3. Synthesis of penipacid A (**6**) from *N*-aminoanthranilic acid

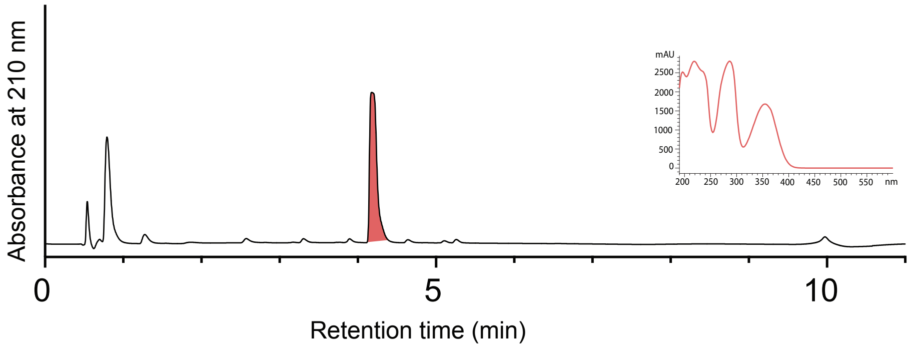

Figure S8. HPLC-DAD (210 nm) chromatogram for the purified penipacid A (highlighted peak, retention time 4.17 min) and UV vis spectrum inset using Agilent Zorbax SB-C_8 column (2.1 × 150 mm, 3.5 mm) running 0.8 mL/min gradient elution from 90% H_2O/MeCN to 100% MeCN (with constant 0.05% formic acid modifier) over 6.5 min.

Table S1. 1D NMR (600 MHz, $CDCl_3$) data for penipacid A (**6**)

Position	penipacid A (**6**)		Reported penipacid A[1] (**1**)	
	δ_H, mult (*J* in Hz)	δ_C	δ_H, mult (*J* in Hz)	δ_C
1	-	108.4	-	108.4
2	-	148.7	-	148.3
3	7.40, d (*8.1)*	113.3	7.50, br d *(8.2)*	113.1
4	7.43, dd *(8.1, 0.9)*	135.9	7.45, td *(8.2, 1.0)*	135.7
5	6.74, dd *(8.0, 0.9)*	117.7	6.76, br t *(7.9)*	117.5
6	7.94, dd *(8.0, 1.2)*	132.0	7.97, dd *(7.9, 0.8)*	131.8
1′-CH_3	1.97, s	18.1	2.00, s	17.9
2′	-	148.5	-	148.4
3′	2.50, s	49.9	2.53, s	49.7
4′	-	70.7	-	70.8
5′-CH_3	1.31, s	29.7	1.35, s	29.4
6′-CH_3	1.31, s	29.7	1.35, s	29.4
1-CO_2H	ND	172.6	-	172.9
2-NH	10.59, s	-	10.63, s	-

*ND not detected

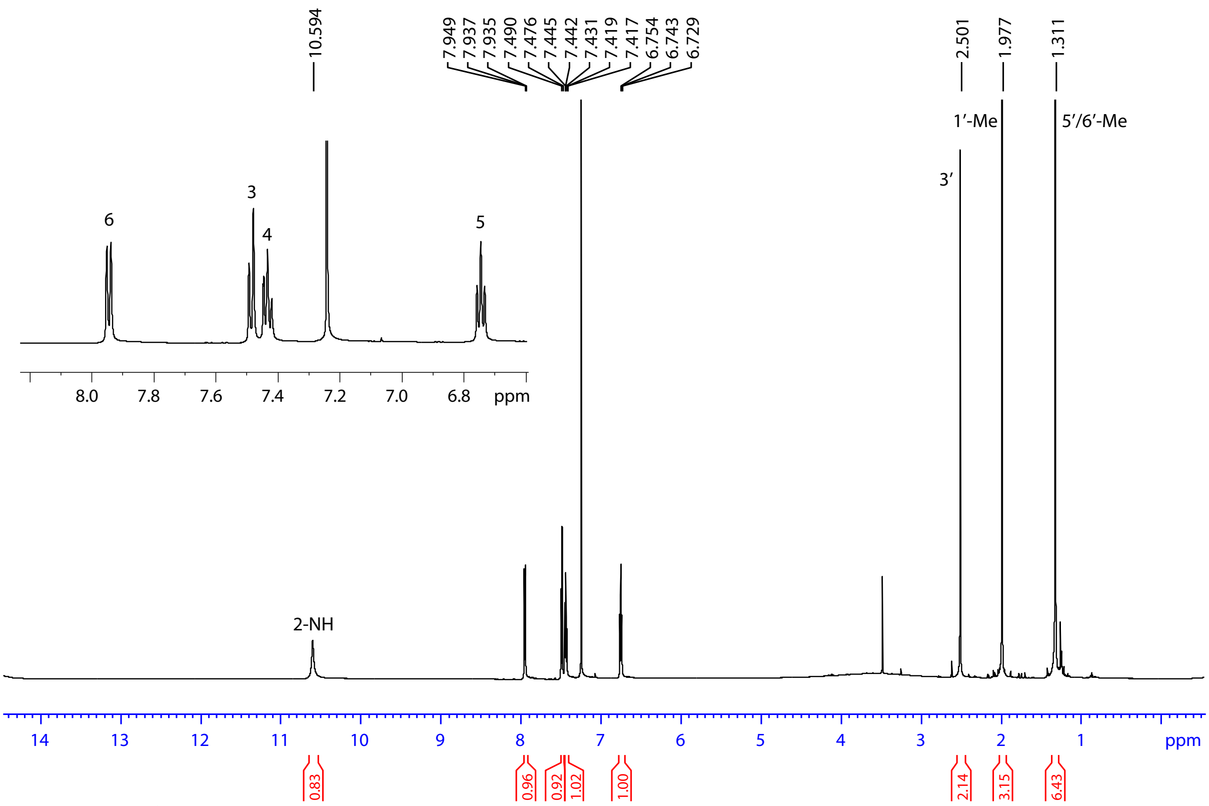

Figure S9. ^{1}H NMR (600 MHz, $CDCl_3$) for penipacid A (**6**)

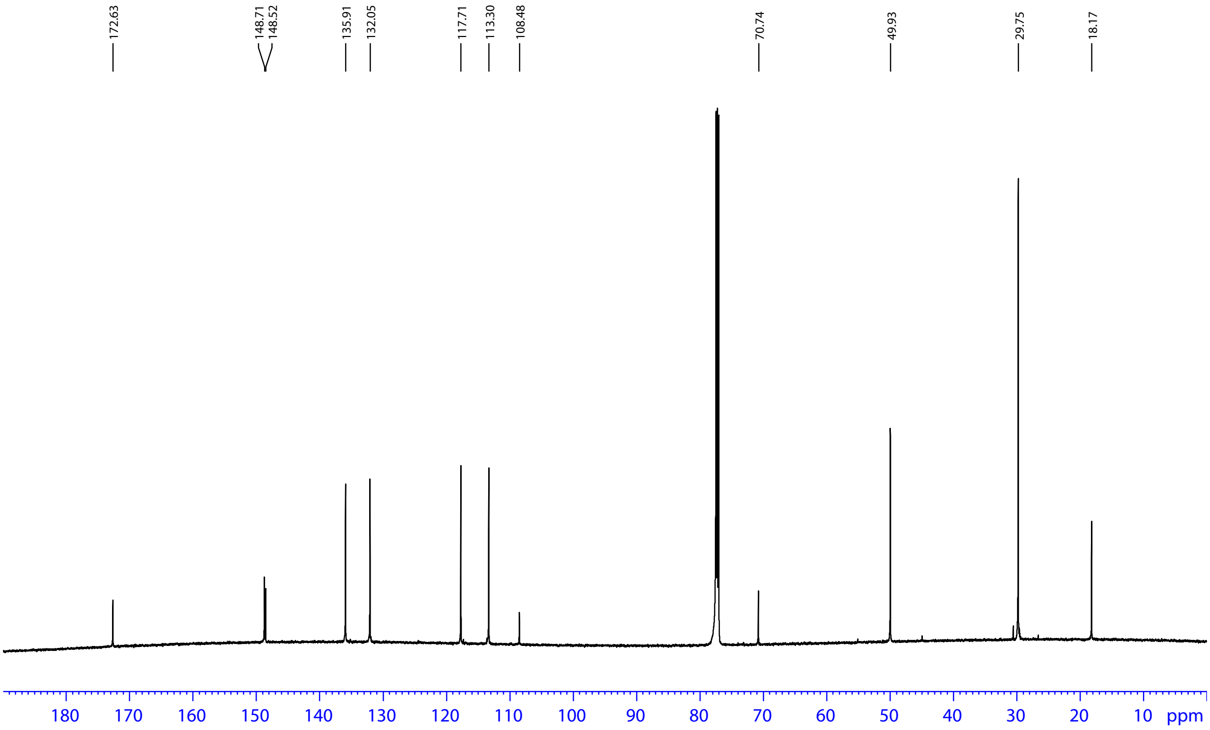

Figure S10. ^{13}C (150 MHz, $CDCl_3$) spectrum for penipacid A (**6**)

Mass Spectrum Molecular Formula Report

Analysis Info

Analysis Name	D:\Data\s.kankanamge\penipacid_A_SP24_25.d	Acquisition Date	9/15/2021 2:53:06 PM
Method	tune-med_AP.m	Operator	a.salim
Sample Name	penipacid_A_SP24_25	Instrument / Ser#	micrOTOF 213750.00232
Comment			

Acquisition Parameter

Source Type	ESI	Ion Polarity	Positive	Set Nebulizer	0.8 Bar
Focus	Not active			Set Dry Heater	180 °C
Scan Begin	100 m/z	Set Capillary	4500 V	Set Dry Gas	5.0 l/min
Scan End	1000 m/z	Set End Plate Offset	-500 V	Set Divert Valve	Source

Generate Molecular Formula Parameter

Formula, min.		
Formula, max.		
Measured m/z	Tolerance	Charge
Check Valence	Minimum	Maximum
Nirogen Rule	Electron Configuration	
Filter H/C Ratio	Minimum	Maximum
Estimate Carbon		

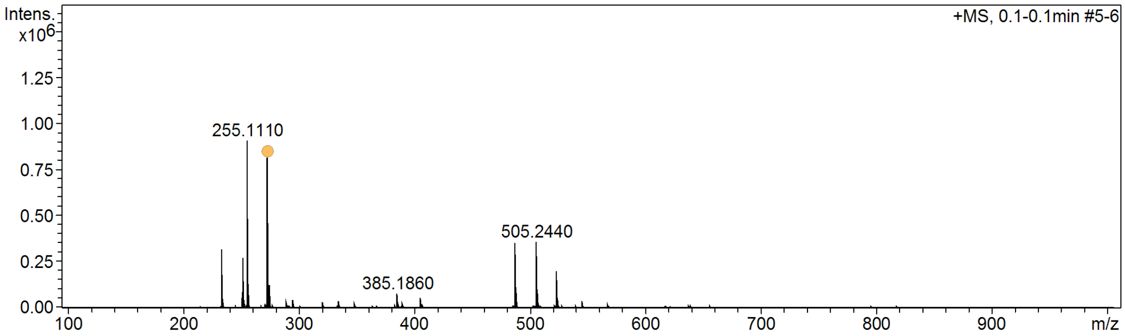

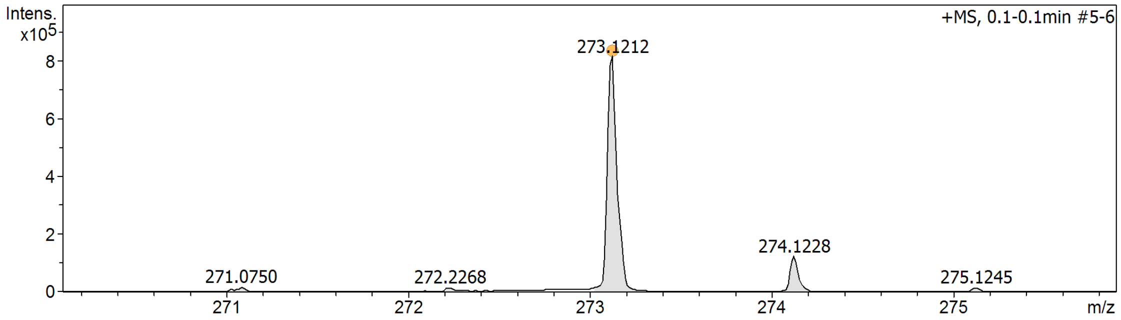

Meas. m/z	#	Ion Formula	m/z	err [ppm]	mSigma	# Sigma	Score	rdb	e⁻ Conf	N-Rule
273.1212	1	C13H18N2NaO3	273.1210	-1.0	1.5	1	100.00	5.5	even	ok

Figure S11. HRMS for penipacid A (**6**)

5 Synthesis of penipacid B (7)

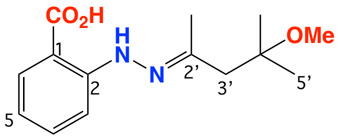

Penipacid B was synthesized by reacting N-aminoanthranilic acid (45 mg, 0.3 mmol) with diacetone alcohol methyl ether (0.3 mmol, 39 mg, 43.8 μL). The reaction mixture was stirred for 5 min at room temperature. The reaction mixture (139 mg) was purified using the semi-preparative reversed phase HPLC (column Zorbax C_8 Eclipse, 9.4 × 250 mm, 5 μm, 65 % MeCN modified with 0.1% TFA, 3 mL/min isocartic, over 20 min) to yield penipacid B in a quantitative yield as yellow oil; R_f: 0.61 (10% MeOH/DCM and the spot was visualized by UV at 254 nm). HRESI(+)MS calculated for $C_{14}H_{20}N_2O_3Na$ 287.1366, found 287.1371.

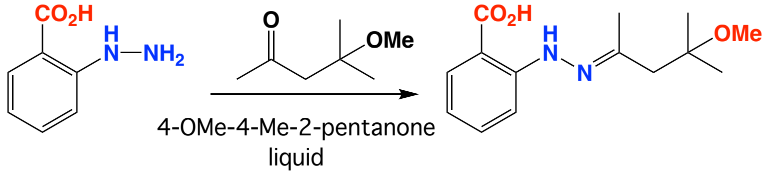

Scheme S4. Synthesis of penipacid B (**7**) from *N*-aminoanthranilic acid

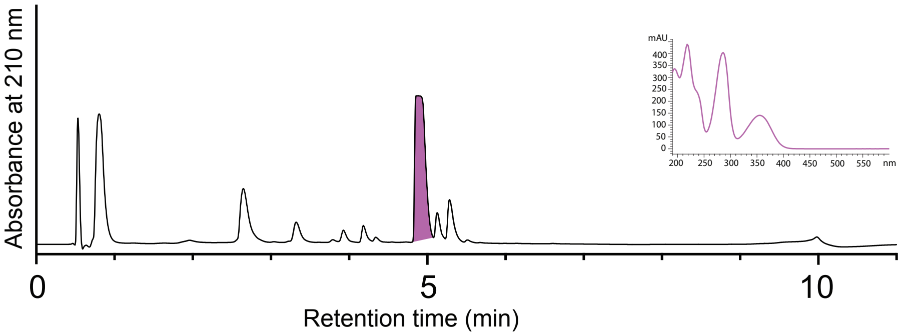

Figure S12. HPLC-DAD (210 nm) chromatogram for the purified penipacid B (highlighted peak) and UV vis spectrum inset, retention time 4.87 min using Agilent Zorbax SB-C_8 column (2.1 × 150 mm, 3.5 mm) running 0.8 mL/min gradient elution from 90% H_2O/MeCN to 100% MeCN (with constant 0.05% formic acid modifier) over 6.5 min.

Table S2. 1D NMR (600 MHz, methanol-d_4) data for penipacid B (**7**)

**Position**	penipacid B (7)		Reported penipacid B[1] (**2**)	
	δ_H, mult (*J* in Hz)	δ_C	δ_H, mult (*J* in Hz)	δ_C
1	-	110.7		111.1
2	-	149.8		149.7
3	7.58, d (*8.5*)	114.2	7.58, br d *(8.5)*	114.1
4	7.39, dd *(8.5, 1.4)*	135.9	7.38, td *(8.5, 1.4)*	135.2
5	6.74, dd *(8.5, 1.1)*	118.0	6.70, br t *(8.0)*	117.9
6	7.89, br d *(7.9)*	132.6	7.89, dd *(8.0, 1.3)*	132.5
1′-CH_3	1.97, s	17.4	1.97, s	17.3
2′	-	148.1		147.9
3′	2.53, s	49.1[a]	2.53, s	48.7
4′	-	76.9		76.8
5′-CH_3	1.23, s	25.7	1.23, s	25.6
6′-CH_3	1.23, s	25.7	1.23, s	25.6
1-CO_2H	ND	172.1		172.3
7′-OMe	3.28, s	49.8	3.27, s	49.0
2-NH	ND	-		

*ND not detected, [a] obscured by MeOH-d_4 signal

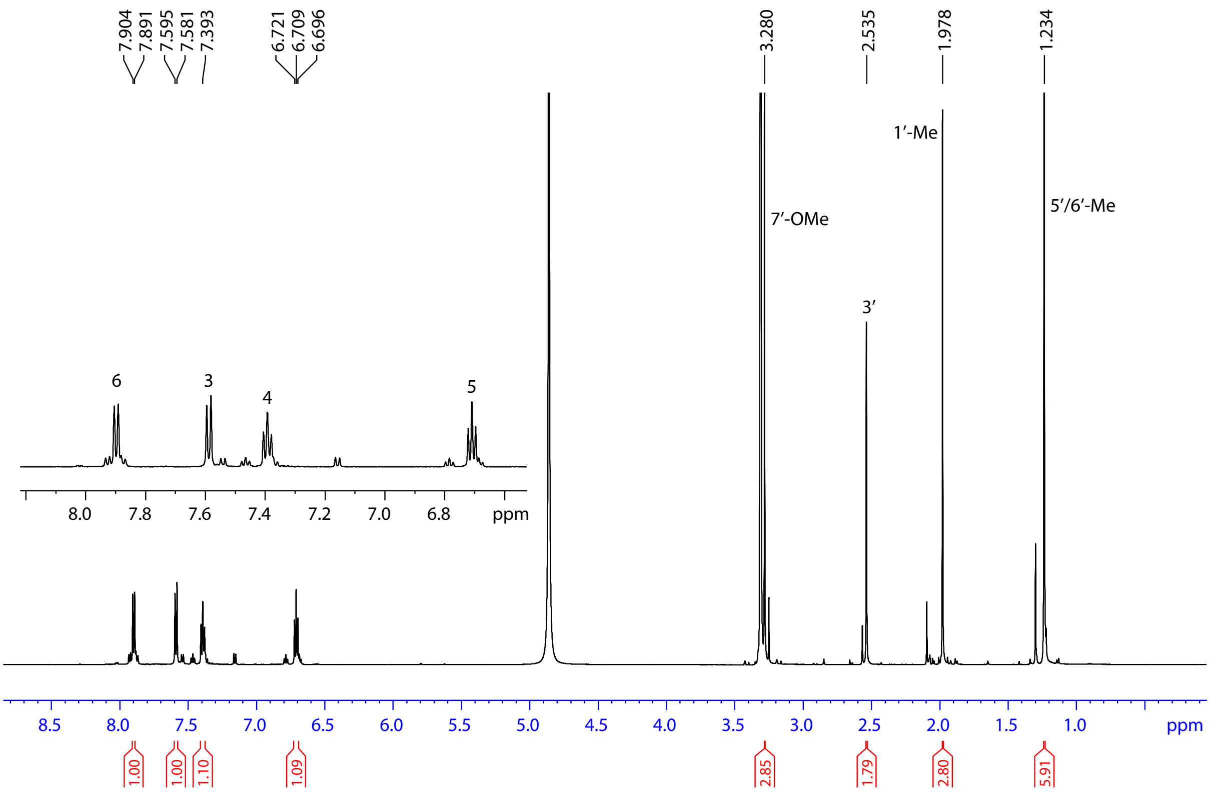

Figure S13. ^{1}H NMR (600 MHz, methanol-d_4) for penicpacid B (**7**)

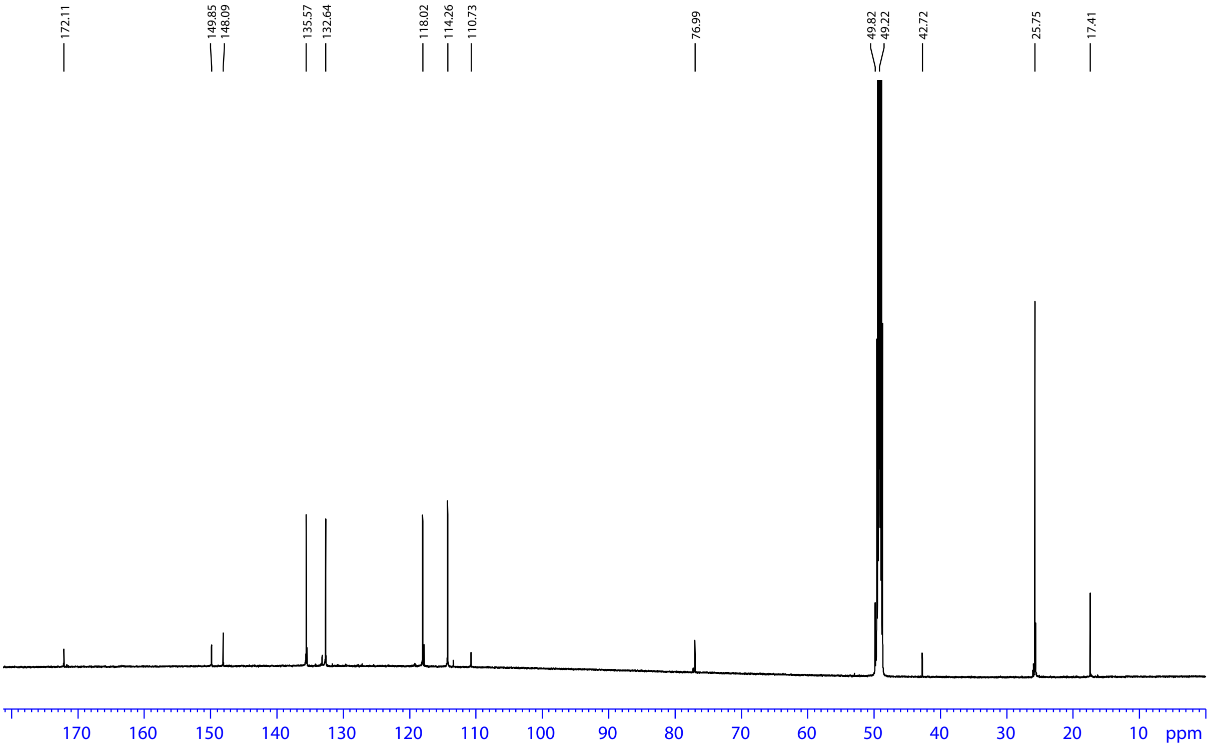

Figure S14. ^{13}C (150 MHz, methanol-d_4) spectrum for penipacid B (**7**)

Mass Spectrum Molecular Formula Report

Analysis Info

Analysis Name	D:\Data\s.kankanamge\penipacid_B_SP13_15.d	Acquisition Date	9/15/2021 2:20:38 PM
Method	tune-med_AP.m	Operator	a.salim
Sample Name	penipacid_B_SP13_15	Instrument / Ser#	micrOTOF 213750.00232
Comment			

Acquisition Parameter

Source Type	ESI	Ion Polarity	Positive	Set Nebulizer	0.8 Bar
Focus	Not active			Set Dry Heater	180 °C
Scan Begin	100 m/z	Set Capillary	4500 V	Set Dry Gas	5.0 l/min
Scan End	1000 m/z	Set End Plate Offset	-500 V	Set Divert Valve	Source

Generate Molecular Formula Parameter

Formula, min.
Formula, max.
Measured m/z — Tolerance — Charge
Check Valence — Minimum — Maximum
Nirogen Rule — Electron Configuration
Filter H/C Ratio — Minimum — Maximum
Estimate Carbon

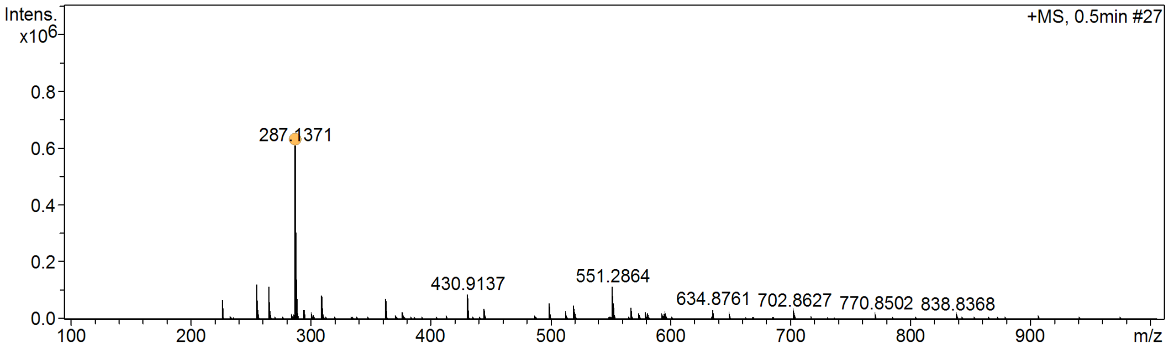

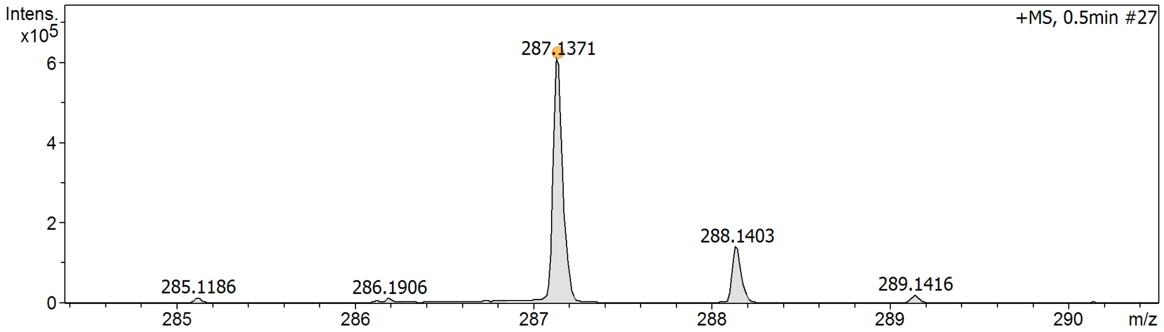

Meas. m/z	#	Ion Formula	m/z	err [ppm]	mSigma	# Sigma	Score	rdb	e¯ Conf	N-Rule
287.1371	1	C14H20N2NaO3	287.1366	1.8	39.8	1	100.00	5.5	even	ok

Figure S15. HRMS for penipacid B (**7**)

6 Synthesis of penipacid C (8)

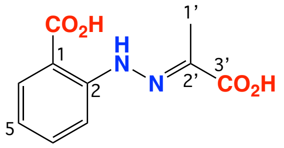

Penipacid C was synthesized by reacting *N*-aminoanthranilic acid (45 mg, 0.3 mmol) with pyruvic acid (0.2 mmol, 26 mg) in MeOH (200 µL). The reaction mixture was stirred for 10 min at room temperature. The reaction mixture (70 mg) was purified using the semi-preparative reversed phase HPLC (column Zorbax C_8, 9.4 × 250 mm, 5 µm, 0 - 55 % MeCN modified with 0.1% TFA, 3 mL/min, over 30 min) to yield penipacid C in a quantitative yield as yellow oil; R_f: 0.43 (10% MeOH/DCM and the spot was visualized by UV at 254 nm). HRESI(+)MS calculated for $C_{10}H_{10}N_2O_4Na$ 245.0533, found 245.0539.

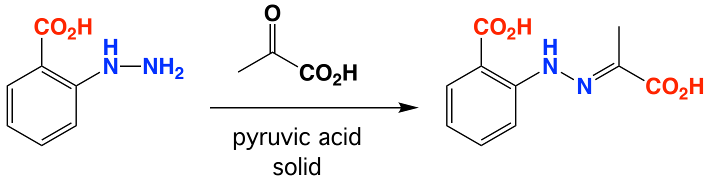

Scheme S5. Synthesis of penipacid C (**8**) from *N*-aminoanthranilic acid

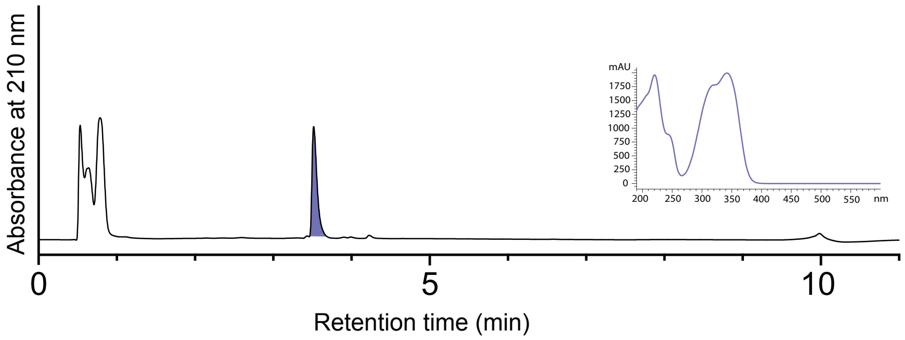

Figure S16. HPLC-DAD (210 nm) chromatogram for the purified penipacid C (highlighted peak) and UV vis spectrum inset, retention time 3.51 min using Agilent Zorbax SB-C_8 column (2.1 × 150 mm, 3.5 mm) running 0.8 mL/min gradient elution from 90% H_2O/MeCN to 100% MeCN (with constant 0.05% formic acid modifier) over 6.5 min.

Table S3. 1D NMR (600 MHz, DMSO-d_6) data for penipacid C (**8**)

Position	penipacid C (**8**)		Reported penipacid C[1] (**3**)	
	δ_H, mult (*J* in Hz)	δ_C	δ_H, mult (*J* in Hz)	δ_C
1	-	112.4	-	112.4
2	-	145.9	-	146.4
3	7.82, d *(8.3)*	113.7	7.81, d *(8.4)*	114.3
4	7.54, dd *(8.0, 1.4)*	134.3	7.55, t *(7.4)*	135.0
5	6.94, dd *(7.9, 1.4)*	119.7	6.94, t *(7.4)*	120.2
6	7.90, dd *(7.9, 1.4)*	131.1	7.89, d *(7.9)*	131.6
1′-CH_3	2.04, s	10.9	2.04, s	11.4
2′	-	135.7	-	136.5
3′- CO_2H	12.38, br s	165.8	NR	166.2
1- CO_2H	ND	169.9	NR	170.4
2-NH	11.49, s	-	11.36, s	-

*ND not detected; NR not reported

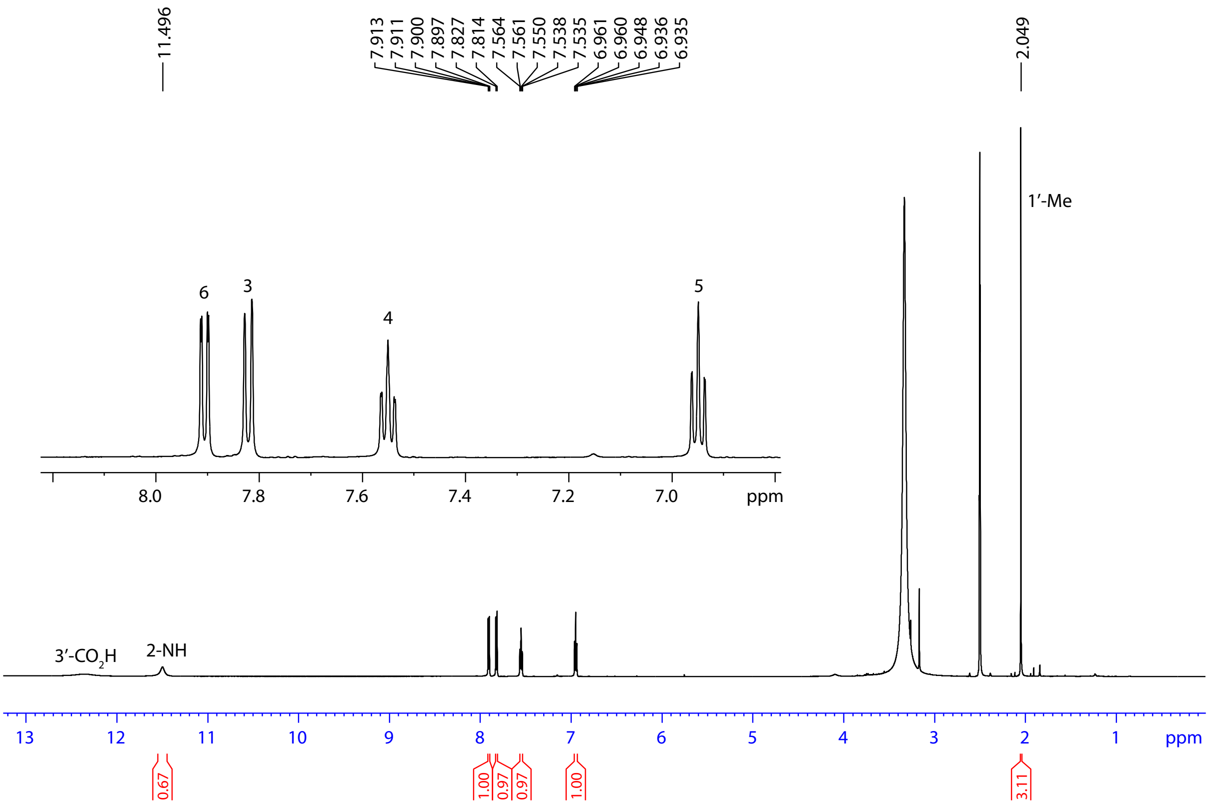

Figure S17. ^{1}H NMR (600 MHz, DMSO-d_6) for penipacid C (**8**)

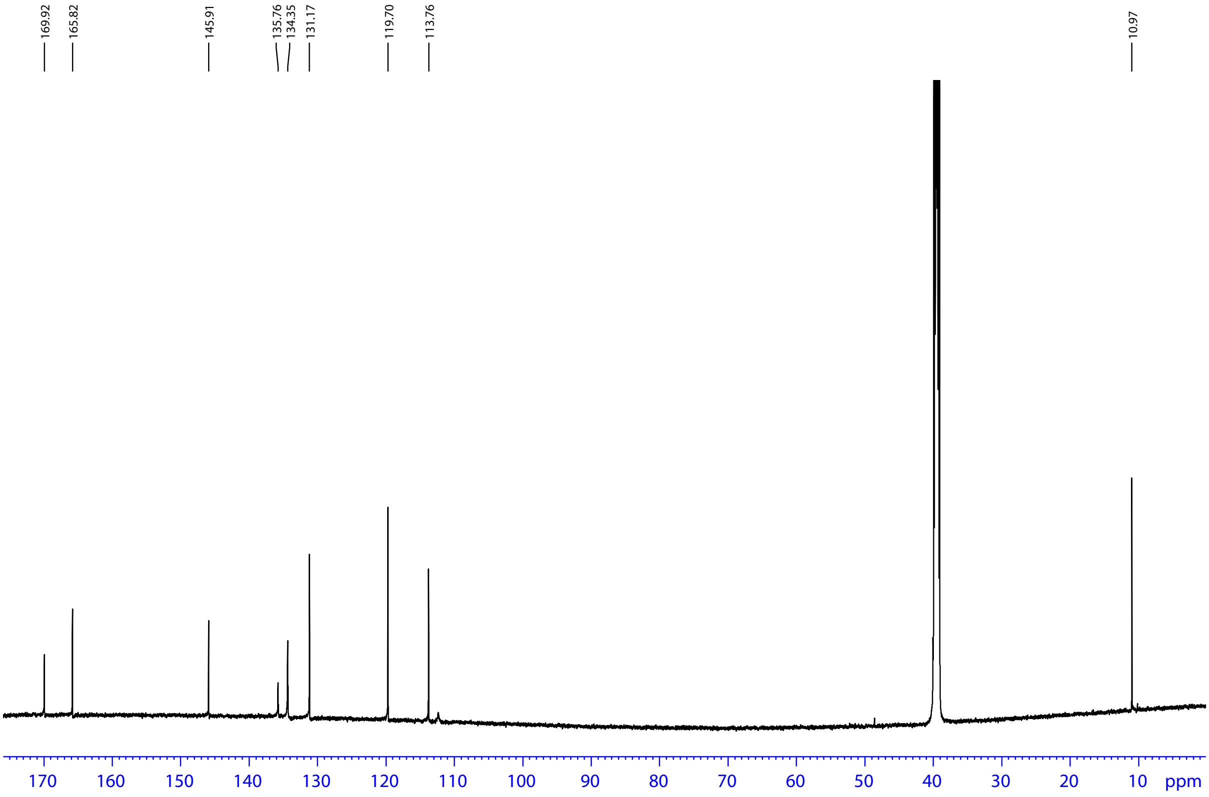

Figure S18. ^{13}C (150 MHz, DMSO-d_6) for penipacid C (**8**)

Mass Spectrum Molecular Formula Report

Analysis Info

Analysis Name	D:\Data\s.kankanamge\penipacid_C_SP16.d	Acquisition Date	9/15/2021 2:27:29 PM
Method	tune-med_AP.m	Operator	a.salim
Sample Name	penipacid_C_SP16	Instrument / Ser#	micrOTOF 213750.00232
Comment			

Acquisition Parameter

Source Type	ESI	Ion Polarity	Positive	Set Nebulizer	0.8 Bar
Focus	Not active			Set Dry Heater	180 °C
Scan Begin	100 m/z	Set Capillary	4500 V	Set Dry Gas	5.0 l/min
Scan End	1000 m/z	Set End Plate Offset	-500 V	Set Divert Valve	Source

Generate Molecular Formula Parameter

Formula, min.		
Formula, max.		
Measured m/z	Tolerance	Charge
Check Valence	Minimum	Maximum
Nirogen Rule	Electron Configuration	
Filter H/C Ratio	Minimum	Maximum
Estimate Carbon		

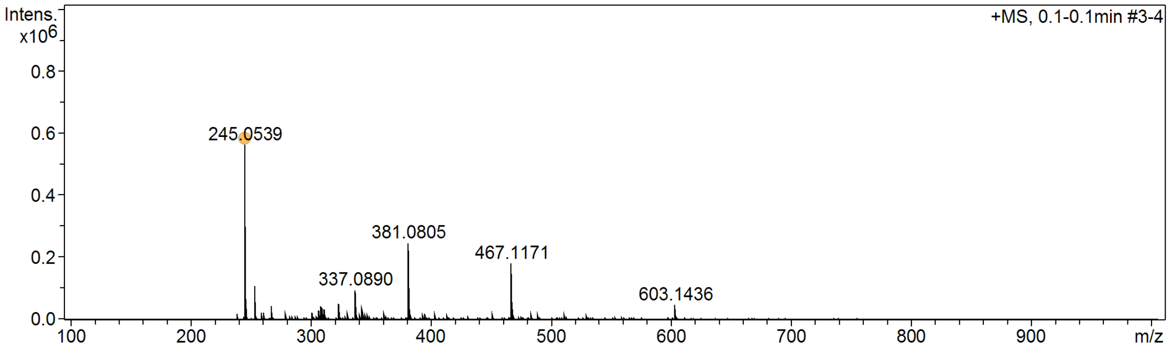

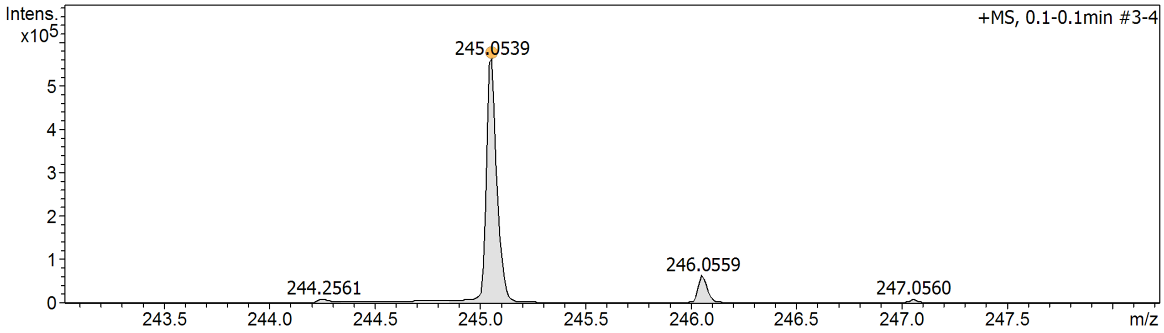

Meas. m/z	#	Ion Formula	m/z	err [ppm]	mSigma	# Sigma	Score	rdb	e⁻ Conf	N-Rule
245.0539	1	C10H10N2NaO4	245.0533	-2.4	3.0	1	100.00	6.5	even	ok
	2	C11H6N6Na	245.0546	-3.1	16.9	2	70.88	11.5	even	ok

Figure S19. HRMS for penipacid C (**8**)

7 Synthesis of penipacid D (9)

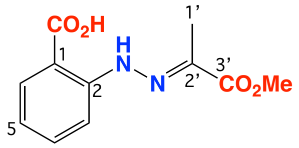

Penipacid D was synthesized by reacting *N*-aminoanthranilic acid (45 mg, 0.3 mmol) with methyl pyruvate (0.3 mmol, 30.6 mg, 27 μL). The reaction mixture was stirred for 10 min at room temperature. The reaction mixture (102 mg) was purified using the semi-preparative reversed phase HPLC (column Zorbax C_8, 9.4 × 250 mm, 5 μm, 0 - 60 % MeCN modified with 0.1% TFA, 3 mL/min, over 30 min) to yield penipacid D in a quantitative yield as yellow oil; R_f: 0.29 (10% MeOH/DCM and the spot was visualized by UV at 254 nm). HRESI(+)MS calculated for $C_{11}H_{12}N_2O_4Na$ 259.0689, found 259.0696.

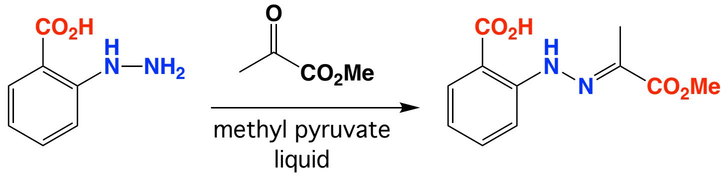

Scheme S6. Synthesis of penipacid D (**9**) from *N*-aminoanthranilic acid

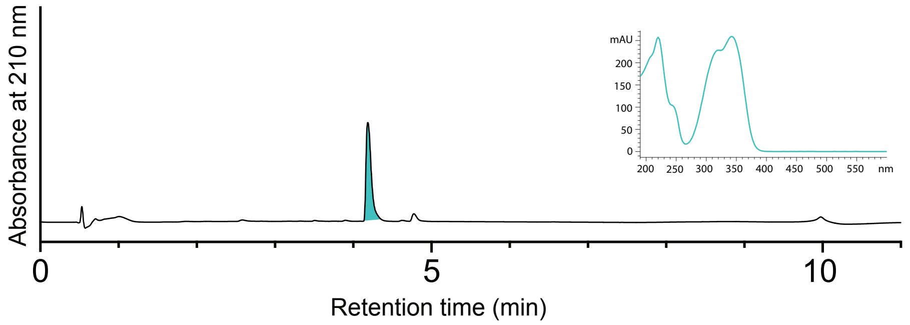

Figure S20. HPLC-DAD (210 nm) chromatogram for the purified penipacid D (highlighted peak) and UV vis spectrum inset, retention time 4.18 min using Agilent Zorbax SB-C_8 column (2.1 × 150 mm, 3.5 mm) running 0.8 mL/min gradient elution from 90% H_2O/MeCN to 100% MeCN (with constant 0.05% formic acid modifier) over 6.5 min.

Table S4. 1D NMR (600 MHz, methanol-d_4) data for penipacid D (**9**)

**Position**	penipacid D (**9**)		Reported penipacid D[1] (**4**)	
	δ_H, mult (*J* in Hz)	δ_C	δ_H, mult (*J* in Hz)	δ_C
1	-	113.9	-	112.1
2	-	147.7	-	147.5
3	7.82, d *(8.4)*	115.2	7.80, d *(8.4)*	115.0
4	7.50, dd *(8.4, 1.2)*	135.5	7.48, t *(7.5)*	135.0
5	6.94, dd *(7.9, 1.1)*	121.1	6.93, t *(7.5)*	121.0
6	7.98, dd *(7.8, 1.1)*	132.6	7.98, d *(7.7)*	132.6
1′-CH_3	2.13, s	11.5	2.14, s	11.4
2′	-	135.9	-	135.4
3′- <u>C</u>O_2Me	-	167.4	-	167.4
3′- CO_2<u>Me</u>	3.84, s	52.8	NR	52.7
1- CO_2H	-	172.1	-	172.7
2-NH	-	-	-	-

*NR not reported

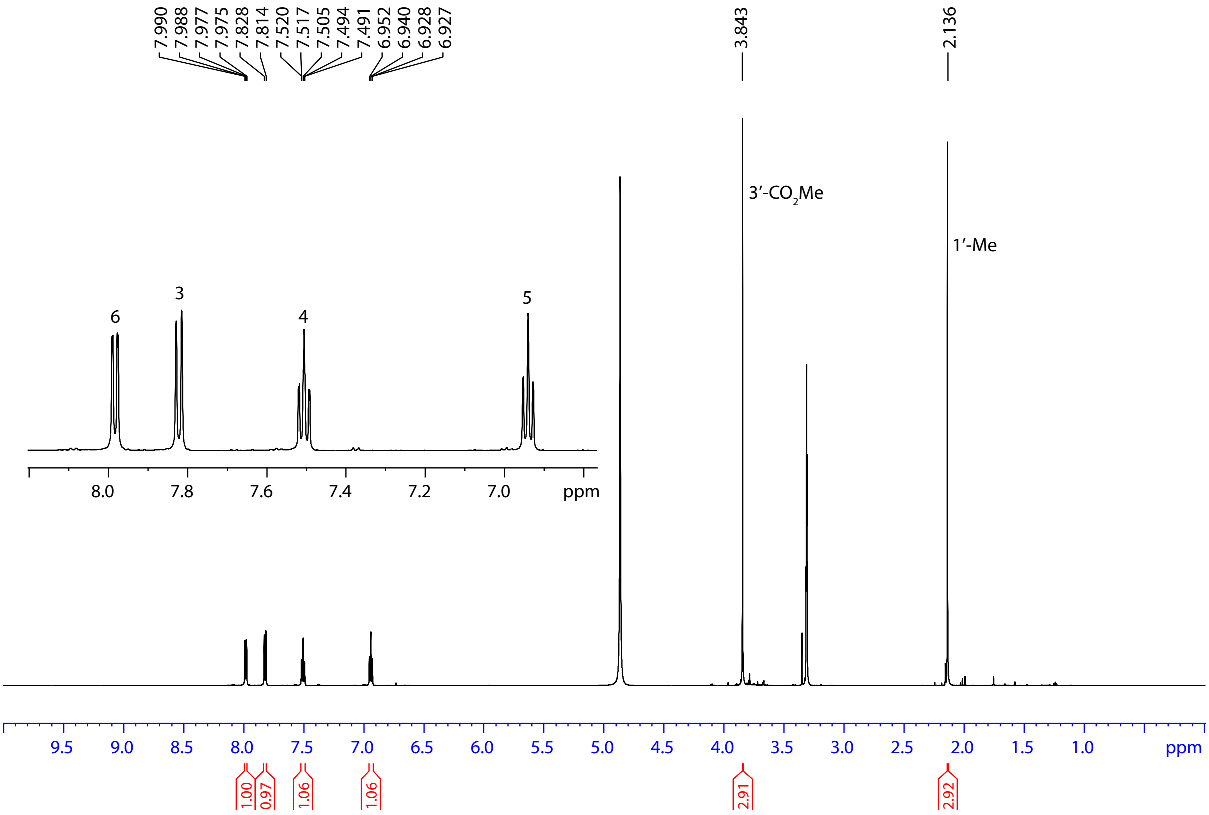

Figure S21. ^{1}H NMR (600 MHz, methanol-*d*4) for penipacid D (**9**)

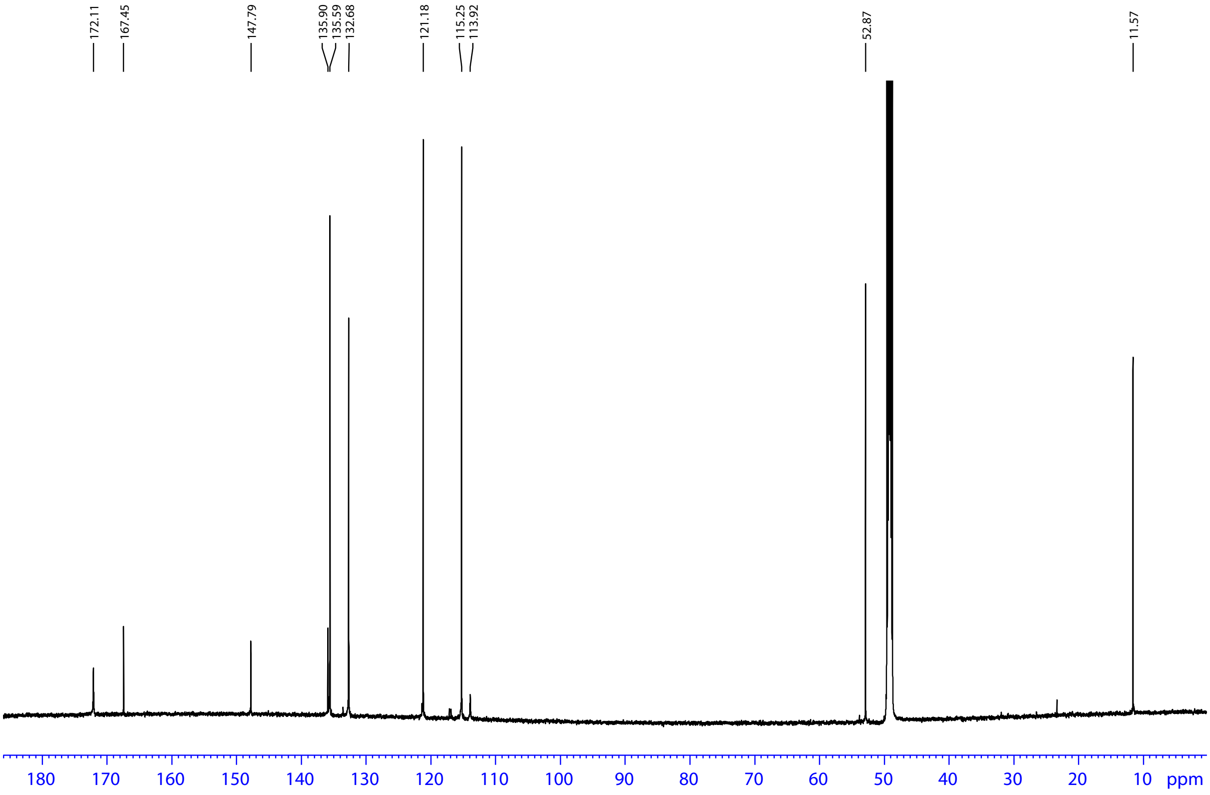

Figure S22. ^{13}C (150 MHz, methanol-*d*4) for penipacid D (**9**)

Mass Spectrum Molecular Formula Report

Analysis Info

Analysis Name	D:\Data\s.kankanamge\penipacid_E_SP8.d	Acquisition Date	9/15/2021 2:34:25 PM
Method	tune-med_AP.m	Operator	a.salim
Sample Name	penipacid_E_SP8	Instrument / Ser#	micrOTOF 213750.00232
Comment			

Acquisition Parameter

Source Type	ESI	Ion Polarity	Positive	Set Nebulizer	0.8 Bar
Focus	Not active			Set Dry Heater	180 °C
Scan Begin	100 m/z	Set Capillary	4500 V	Set Dry Gas	5.0 l/min
Scan End	1000 m/z	Set End Plate Offset	-500 V	Set Divert Valve	Source

Generate Molecular Formula Parameter

Formula, min.		
Formula, max.		
Measured m/z	Tolerance	Charge
Check Valence	Minimum	Maximum
Nirogen Rule	Electron Configuration	
Filter H/C Ratio	Minimum	Maximum
Estimate Carbon		

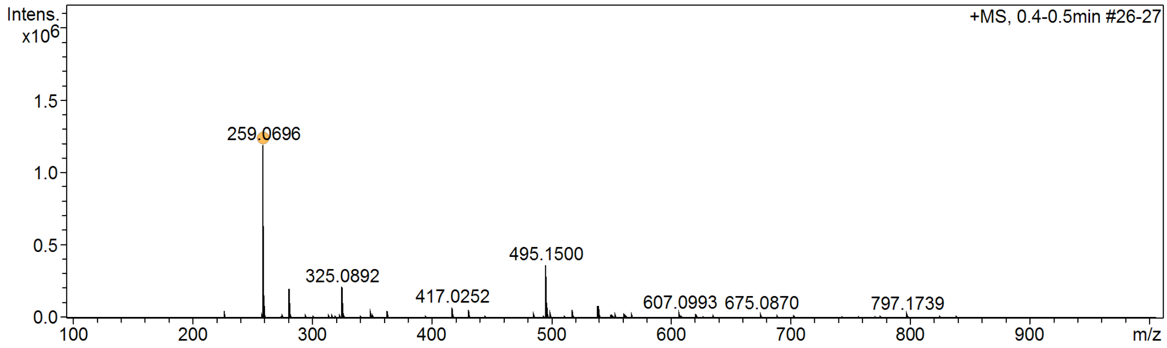

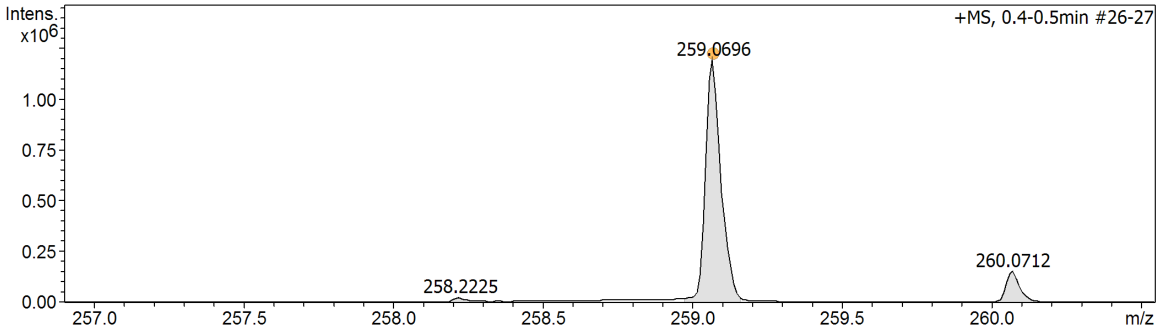

Meas. m/z	#	Ion Formula	m/z	err [ppm]	mSigma	# Sigma	Score	rdb	e⁻ Conf	N-Rule
259.0696	1	C11H12N2NaO4	259.0689	2.6	1.6	1	100.00	6.5	even	ok
	2	C12H8N6Na	259.0703	-2.6	15.1	2	78.84	11.5	even	ok

Figure S23. HRMS for penipacid D (**9**)

8 Synthesis of penipacid E (10)

Penipacid E was synthesized by reacting *N*-aminoanthranilic acid (45 mg, 0.3 mmol, 1 eq) with furfural (0.6 mmol, 40 mg, 50 µL, 2 eq). The reaction mixture was stirred for 10 min at room temperature. The reaction mixture (93 mg) was purified using the semi-preparative reversed phase HPLC (column Zorbax Phenyl, 9.4 × 250 mm, 5 µm, 0 - 65 % MeCN modified with 0.1% TFA, 3 mL/min, over 30 min), to yield penipacid E as yellow oil (0.05 mg, 0.07 %); R_f: 0.4 (10% MeOH/DCM and the spot was visualized by UV at 254 nm). HRESI(+)MS calculated for $C_{12}H_{10}N_2O_3Na$ 2593.0584, found 259.0579.

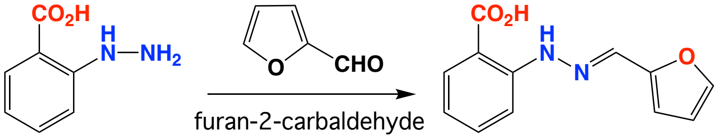

Scheme S7. Synthesis of penipacid E (**10**) from *N*-aminoanthranilic acid

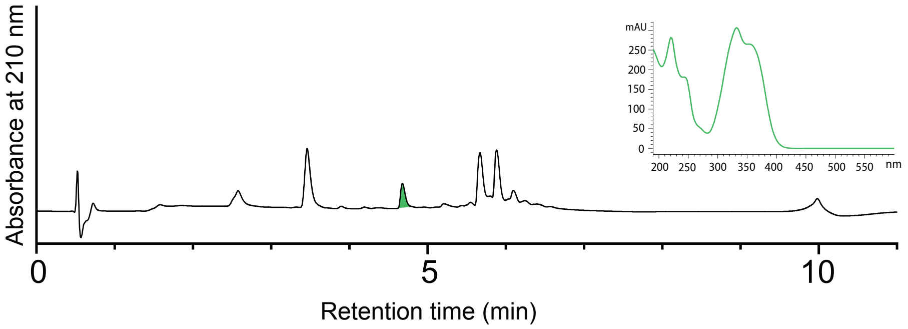

Figure S24. HPLC-DAD (210 nm) chromatogram for the reaction mixture of penipacid E (highlighted peak) and UV vis spectrum inset, retention time 4.67 min using Agilent Zorbax SB-C_8 column (2.1 × 150 mm, 3.5 mm) running 0.8 mL/min gradient elution from 90% H_2O/MeCN to 100% MeCN (with constant 0.05% formic acid modifier) over 6.5 min.

Mass Spectrum Molecular Formula Report

Analysis Info

Analysis Name	D:\Data\s.kankanamge\penipacid_G_SP15.d	Acquisition Date	9/15/2021 2:50:26 PM
Method	tune-med_AP.m	Operator	a.salim
Sample Name	penipacid_G_SP15	Instrument / Ser#	micrOTOF 213750.00232
Comment			

Acquisition Parameter

Source Type	ESI	Ion Polarity	Positive	Set Nebulizer	0.8 Bar
Focus	Not active			Set Dry Heater	180 °C
Scan Begin	100 m/z	Set Capillary	4500 V	Set Dry Gas	5.0 l/min
Scan End	1000 m/z	Set End Plate Offset	-500 V	Set Divert Valve	Source

Generate Molecular Formula Parameter

Formula, min.		
Formula, max.		
Measured m/z	Tolerance	Charge
Check Valence	Minimum	Maximum
Nirogen Rule	Electron Configuration	
Filter H/C Ratio	Minimum	Maximum
Estimate Carbon		

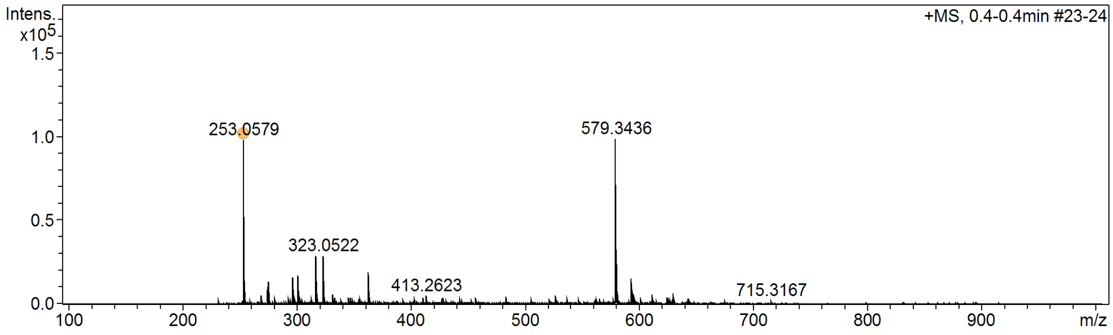

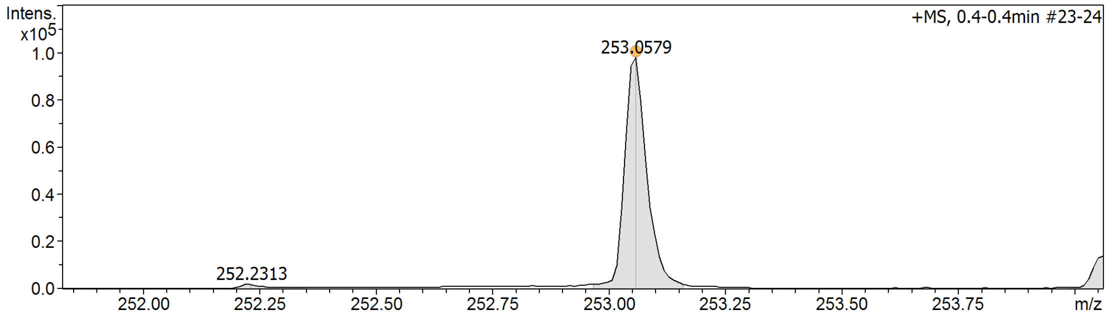

Meas. m/z	#	Ion Formula	m/z	err [ppm]	mSigma	# Sigma	Score	rdb	e⁻ Conf	N-Rule
253.0579	1	C12H10N2NaO3	253.0584	-2.0	2.8	1	100.00	8.5	even	ok

Figure S25. HRMS for penipacid E (**10**)

9 Biological assays

9.1 Antibacterial and antifungal assays

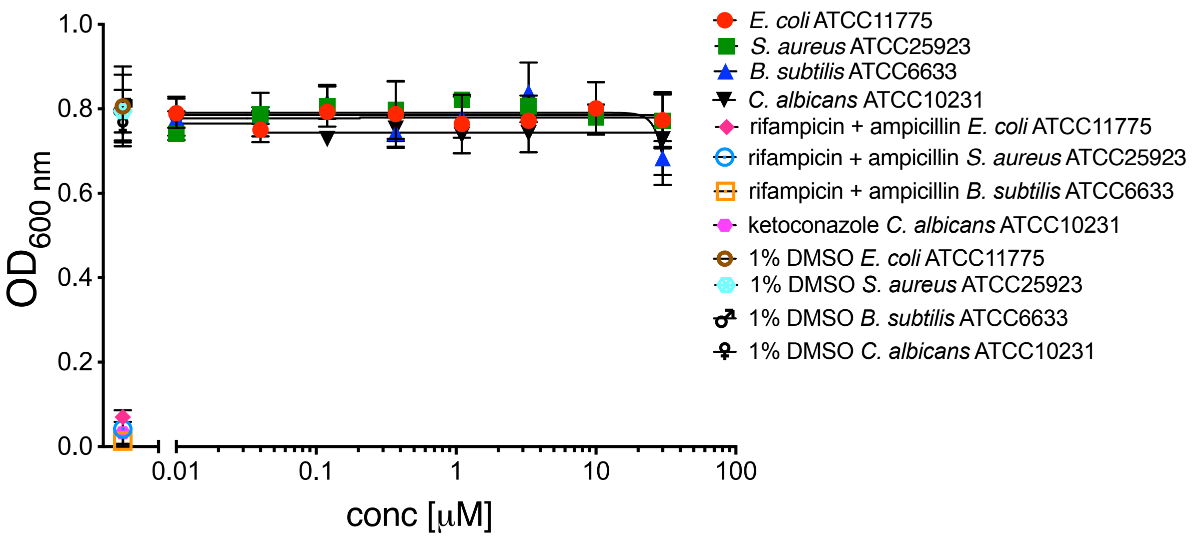

Figure S26. Growth inhibitory activity of *N*-aminoanthranilic acid

9.2 Cytotoxicity (MTT) assay

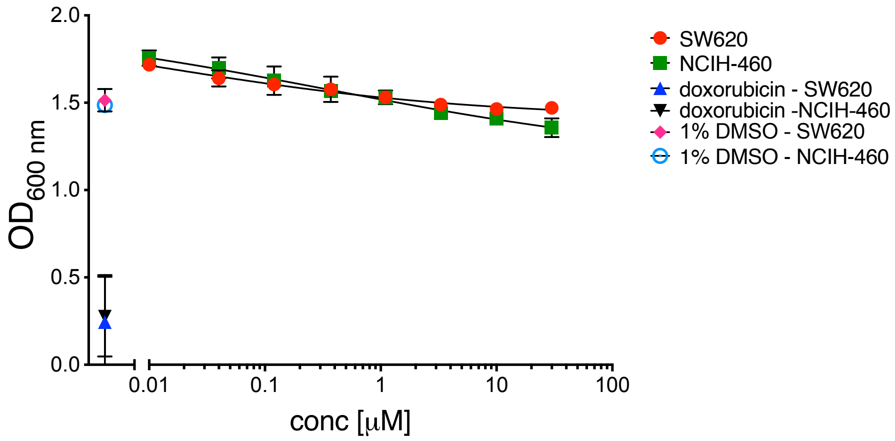

Figure S27. Cytotoxicity of *N*-aminoanthranilic acid against SW620 and NCIH-460. Doxorubicin and 1%DMSO served as positive and negative controls respectively.

Table S5. List of bacterial strains for the activation trials

**Strains**	**Taxonomy**	**Cultivation medium**
CMB-M0139	Unspecified	M1 + 3.3% sea salt
CMB-CA059	*Streptomyces platensis*	IMA
CMB-CA091	*Streptomyces virginiae*	IMA
CMB-PB041	*Streptomyces sp.*	ISP2
CMB-PB042	*Streptomyces sp.*	ISP2
ACM-4200	*Streptomyces hawaiiensis*	ISP2
ACM-4361	*Streptomyces sampsonii*	ISP2
ACM-4129	*Streptomyces eurythermus*	ISP2
ACM-4105	*Streptomyces corchorusii*	ISP2
ACM-4477	*Streptomyces spectabilis*	ISP2
ACM-4279	*Streptomyces niveus*	ISP2

Table S6. List of fungal strains for the activation trials

**Strains**	**Taxonomy**	**Cultivation medium**
CMB-GO296	Unspecified	SDB
CMB-W003	Unspecified	SDB
CMB-GO014	Unspecified	IMA
CMB-GO008	Unspecified	ISP2
CMB-M0339	*Aspergillus sp.*	D400
CMB-NF091	*Talaromyces sp.*	YEME
S4S-00182	Unspecified	ISP2
CMB-MRF324	*Aspergillus sp.*	SDB
CMB-F458	*Scopulariopsis* sp.	M1 + 3.3% sea salt
CMB-MD22	*Penicillium* sp.	ISP2
CMB-MD14	*Penicillium* sp.	ISP2

10 References

1. Li, C.-S.; Li, X.-M.; Gao, S.-S.; Lu, Y.H.; Wang, B.-G. Cytotoxic anthranilic acid derivatives from deep sea sediment-derived fungus *Penicillium pan*eum SD-44. *Mar. Drugs* **2013**, *11*, 3068–3076.
2. Dang, T.; Suchy, M.; Truong, Y.J.; Oakden, W.; Lam, W.W.; Lazurko, C.; Facey, G.; Stanisz, G.J.; Shuhendler, A.J. Hydrazo-CEST: Hydrazone-dependent chemical exchange saturation transfer magnetic resonance imaging contrast agents. *Chem. Eur. J.* **2018**, *24*, 9148–9156.
